# EM-transcriptomic signature predicts drug response in advanced stages of high-grade serous ovarian carcinoma based on ascites-derived primary cultures

**DOI:** 10.3389/fphar.2024.1363142

**Published:** 2024-03-06

**Authors:** Diana-Roxana Constantinescu, Andrei Sorop, Alina-Veronica Ghionescu, Daniela Lixandru, Vlad Herlea, Nicolae Bacalbasa, Simona Olimpia Dima

**Affiliations:** ^1^ Center of Excellence in Translational Medicine, Fundeni Clinical Institute, Bucharest, Romania; ^2^ University of Medicine and Pharmacy “Carol Davila”, Bucharest, Romania; ^3^ Department of Pathology-Fundeni Clinical Institute, Bucharest, Romania; ^4^ Center of Digestive Diseases and Liver Transplantation, Fundeni Clinical Institute, Bucharest, Romania

**Keywords:** high-grade serous ovarian carcinoma, ascites-derived primary cultures, transcriptomic signature, chemoresistance, epithelial–mesenchymal transition

## Abstract

**Introduction:** High-grade serous ovarian carcinoma (HGSOC) remains a medical challenge despite considerable improvements in the treatment. Unfortunately, over 75% of patients have already metastasized at the time of diagnosis. Advances in understanding the mechanisms underlying how ascites cause chemoresistance are urgently needed to derive novel therapeutic strategies. This study aimed to identify the molecular markers involved in drug sensitivity and highlight the use of ascites as a potential model to investigate HGSOC treatment options.

**Methods:** After conducting an *in silico* analysis, eight epithelial–mesenchymal (EM)-associated genes related to chemoresistance were identified. To evaluate differences in EM-associated genes in HGSOC samples, we analyzed ascites-derived HGSOC primary cell culture (AS), tumor (T), and peritoneal nodule (NP) samples. Moreover, *in vitro* experiments were employed to measure tumor cell proliferation and cell migration in AS, following treatment with doxorubicin (DOX) and cisplatin (CIS) and expression of these markers.

**Results:** Our results showed that AS exhibits a mesenchymal phenotype compared to tumor and peritoneal nodule samples. Moreover, DOX and CIS treatment leads to an invasive-intermediate epithelial-to-mesenchymal transition (EMT) state of the AS by different EM-associated marker expression. For instance, the treatment of AS showed that CDH1 and GATA6 decreased after CIS exposure and increased after DOX treatment. On the contrary, the expression of KRT18 has an opposite pattern.

**Conclusion:** Taken together, our study reports a comprehensive investigation of the EM-associated genes after drug exposure of AS. Exploring ascites and their associated cellular and soluble components is promising for understanding the HGSOC progression and treatment response at a personalized level.

## 1 Introduction

HGSOC is a major cause of gynecological tumor mortality, with approximately 70% out of 200,000 ovarian cancer deaths annually (Bowtell et al., 2015), according to the GLOBOCAN 2020 report (Sung et al., 2021). Using the most recent data available, the age-standardized mortality rate of ovarian cancer in Romania was approximately 2.9 deaths per 100,000 population (Mazidimoradi et al., 2022), World Health Organization (2019).

HGSOC patients are typically asymptomatic in the early stages and are treated with primary debulking surgery (PDS), while chemotherapy is the preferred option for advanced-stage cancer. Carboplatin/cisplatin (CARB/CIS) and paclitaxel (PAX) are first-line chemotherapeutic agents, but the US Food and Drug Administration (FDA) has also approved DOX, gemcitabine (GEM), irinotecan (MM-398), etoposide (ETOP), oxaliplatin (OHP), and 5-fluorouracil (5-FU) (Lheureux et al., 2019; Lisio et al., 2019; Bacalbasa et al., 2020a; Armstrong et al., 2021; Ogundipe et al., 2023). Unfortunately, most patients experience recurrence and acquire resistance to platinum-based agents (Bowtell et al., 2015). The inhibition of apoptosis is the mechanism by which chemoresistant tumor cells are propagated (Kunjachan et al., 2013; Caccuri et al., 2019; Neophytou et al., 2021; Duan et al., 2023); one of the causes is the inhibition of caspase activity, which is due to the changes and mutations in signaling pathways. Mammalian cancer cells will lose their promoters and have no ability to induce apoptosis, causing resistance to cytotoxic drugs (Ghavami et al., 2009; Mohamed et al., 2017; Jan and Chaudhry, 2019; Jiang et al., 2020).

This type of ovarian cancer is characterized by genetic mutations in tumor suppressor genes *TP53*, *BRCA1*, or *BRCA2* and by a specific dissemination mechanism through the body cavity known as transcoelomic metastasis. This mechanism involves rapid growth, disruption of ovarian tumor capsules, and malignant cells spreading into the peritoneal cavity by the ascites fluid, whose role is to offer the tumor microenvironment (Tan et al., 2006; Kurman and Shih I.e., 2008; Shield et al., 2009; The Cancer Genome Atlas Research Network, 2011; Ahmed and Stenvers, 2013; Suh et al., 2014; Kim et al., 2016; Barbolina, 2018; Loret et al., 2019). Tumor formation is driven by “tumor-initiating cells” that exhibit mesenchymal and stem cell features. Activation of EMT induces HGSOC precursor lesion (secretory cell outgrowths, SCOUTS, and serous tubal intraepithelial carcinoma, STIC) by suppressing paired box protein 2 (PAX2), a key molecule in maintaining the differentiation state of oviductal epithelial cells. A critical step in the progression of HGSOC is the migration of STIC cells to the ovary (Chen et al., 2010; Perets et al., 2013; Alwosaibai et al., 2017; Tone, 2017). Moreover, growth factors and hormones are secreted onto the ovarian surface to induce EMT via phosphoinositide-3-kinase/Akt (PI3K/AKT) and mitogen-activated protein kinase/extracellular signal-regulated kinase (MEK/ERK) signaling pathways (Wong and Leung, 2007; Gao et al., 2014; Dean et al., 2017). In the metastatic stage, cancer cells are released from the primary tumor directly into the peritoneal cavity and survive either as single cells or spheroids in the ascites fluid, causing the formation of peritoneal nodules (peritoneal carcinomatosis) and also metastases to distant organs (Tan et al., 2006; Shield et al., 2009; Lengyel, 2010; Bacalbasa et al., 2020b). EMT upregulates α5β1 integrin, which mediates spheroid attachment to the secondary site (Al Habyan et al., 2018; Li et al., 2020). Moreover, Rosso et al. reported higher EMT marker expression in ascites cultures than in tumor cultures, indicating its crucial role in metastatic dissemination (Rosso et al., 2017). Nevertheless, even the mesenchymal-to-epithelial transition (MET) process has been described in the metastatic cascade, as associated with epigenetic abnormalities (Fan et al., 2020). At the cellular level, the partial EMT promotes ascites and metastasis formation in HGSOC (Loret et al., 2019).

Along with EMT markers, the cytokeratin family (KRTs), the most abundant proteins in epithelial cells, is pivotal in maintaining keratinization and differentiation. These have been reported to conserve cell morphology, intracellular transport, and signal transduction (Jacob et al., 2018; Zhang et al., 2020). In many cancers, such as ovarian, breast, and lung cancers, KRTs could be used as prognosis and tumorigenesis status markers (Blobel et al., 1984; Shao et al., 2012; Communal et al., 2021). Their expression has been associated with a high grade of malignancy and an increase in the migratory capability of cancer cells (Zhang et al., 2020).

An accumulating number of studies have highlighted the molecular heterogeneity of ovarian cancer, suggesting the need for personalized treatment approaches, including the establishment of ascites-derived cultures (RL et al., 2014; Kim et al., 2016; Penet et al., 2018; Uno et al., 2022). The ascites contains detached cancer cells, extracellular vesicles (EVs), tumor-associated macrophages (TAMs), and host cells, together promoting proliferation, drug resistance, or metastasis (Zhang et al., 2018). Hence, ascites reveals essential information about the underlying malignancy before resection that includes molecular mechanisms and profiles (Kipps et al., 2013). Considering these aspects, ascites presents a chance to design a treatment plan for patients with ovarian cancer by its potential use as a liquid biopsy substrate for exploring novel therapeutic targets (Latifi et al., 2012; Ahmed and Stenvers, 2013; Ford et al., 2020).

This study aimed to display the differences between the peritoneal nodule, primary tumor, and AS, the three major types of biological material derived from patients with HGSOC. We also conducted a computational target molecule prediction involved in chemoresistance. After *in vitro* drug testing of ascites-derived HGSOC primary cell culture, we reported a comprehensive investigation of the EM-associated genes.

## 2 Materials and methods

### 2.1 *In silico* analysis of available databases

#### 2.1.1 The acquisition of mRNA expression datasets

The transcriptome profiles and relevant clinical information on patients with ovarian serous cystadenocarcinoma (OV) have been derived from The Cancer Genome Atlas (TCGA) (https://portal.gdc.cancer.gov/), and normal human ovarian samples have been obtained from The Genotype-Tissue Expression (GTEx) (https://www.gtexportal.org/home/datasets), using the TCGAbiolinks package (Colaprico et al., 2016) in the R program (version 4.3.0). Then, both RNA sequencing (RNA-seq) data (displayed as raw counts) were combined with batch normalization using the R package “sva” (Zhang et al., 2023). TCGA-OV dataset included 421 tumor samples, and the GTEx included 108 normal ovarian samples. TCGA and GTEx expression data were normalized in transcripts per million (TPM) format. The prognostic information on TCGA-OV samples was acquired from the UCSC Xena database (https://xenabrowser.net/).

#### 2.1.2 Differential gene expression analysis

The differentially expressed protein-coding genes (DEGs) from tumor and normal ovarian tissues were generated using the DESeq2 (version 1.40.2) package of R software (Love et al., 2014). DEGs were selected based on a false discovery rate (FDR) p. adj< 0.01 and absolute logFC (fold change) ≥2. Principal component analysis was performed to examine relationships between tumoral and normal samples using a variance stabilizing transformation function to the count data (Wu et al., 2020) (Supplementary Figure S1). Next, the EnhancedVolcano (version 1.18.0) R package was used to visualize the results of differential expression analyses (Supplementary Figure S2). GO enrichment analysis of a gene set was performed using clusterProfiler (version 4.8.2) R package, and as a result, the significantly enriched GO terms were those with adjusted *p*-value <0.05.

Furthermore, we used the keywords “(drug-resistant) AND (EMT) AND (keratin) AND (ovarian cancer)” to search in the GeneCards (https://www.genecards.org/) database and obtained 1,265 drug resistance-EMT-keratin-related protein-coding genes. After the published literature was reviewed, a list of eight EM-associated genes, namely, five EMT—cadherin 1 (CDH1), cadherin 2 (CDH2), epithelial cell adhesion molecule (EPCAM), vimentin (VIM), GATA-binding protein 6 (GATA6) and three keratin markers—keratin 7 (KRT7), keratin 18 (KRT18), and keratin 19 (KRT19), was further explored. To construct and analyze the protein–protein interactions of our EM-associated genes, we submitted them to STRING (version 12, https://string-db.org/), a web-based open-access software tool.

#### 2.1.3 Validation of EM-associated genes related to chemoresistance

In addition, the mRNA expression profile from HGSOC patients and ovarian cancer cell lines treated with platinum drugs was downloaded from the Gene Expression Omnibus (GEO, http://www.ncbi.nlm.nih.gov/geo/) database using the GEOquery R packages, which were GSE227100, GSE58470, and GSE98559.

These three above-mentioned datasets were, respectively, derived from *Homo sapiens* mRNA data, using GPL24676 Illumina NovaSeq 6000, GPL16791 Illumina HiSeq 2500,and GPL6947 Illumina HumanHT-12 V3.0 expression bead chip.

The GSE227100 included a total of 24 HGSOC patients diagnosed with FIGO Stage III/IV, from which we obtained solid tumor samples before ((pre-C/T) and after (post-C/T) completion of six cycles of CARB and Taxol combination chemotherapy. We used GSE98559 with two experimental groups, SKOV3 wild-type cells and SKOV3 cisplatin-resistant cells, each having two biological replicate samples analyzed (four samples). We also explored three ovarian carcinoma cell lines: the parental cisplatin-sensitive IGROV-1 cell line and two platinum-resistant variants (IGROV-1 CIS/IGROV-1 OHP); each cell line had three independent samples analyzed (nine samples altogether) from GSE58470. Hierarchical clustering was performed with the pheatmap (version 1.0.12) R package using “Euclidean” clustering to calculate row distances and “complete” the agglomeration method (the distance between the most distant elements in each cluster).

### 2.2 Patient inclusion and sample collection

The study included a total of 12 AS, 9 T, and 7 NP from 12 HGSOC patients who underwent surgical resection at the Fundeni Clinical Institute between 2019 and 2021. The study was approved by the Ethics Committee of the Fundeni Clinical Institute (52496/06.12.2018). All the experiments were conducted following the Helsinki Declaration and obeying ethical principles for medical research on human subjects. Collected clinical and pathologic data include age, TNM stage, differentiation degree, tumor size (cm), serum tumor biomarkers (CA125 (ng/mL), CA15-3 (ng/mL), CEA (ng/mL), and CA19-9 (U/mL)), and overall survival (months). Histopathological results and the tumor grade were determined by the pathologist, according to the International Federation of Gynecology and Obstetrics (FIGO) classification. The cohort had not received preoperative chemotherapy at the time of debulking surgery. NP and T tissue samples were collected during surgery—in a stabilizing solution RNAlater (Sigma, St. Louis, MO)—then cryopreserved—using the snap-frozen method—and stored at −80 °C until further analysis.

### 2.3 Cell culture

#### 2.3.1 SKOV3 cells

The human ovarian cancer cell line SKOV3 was purchased from the European Collection of Authenticated Cell Cultures (ECACC). SKOV3 was cultured in McCoy’s 5A medium (modified), w: L-glutamine, w: 2.2 g/L NaHCO_3_ (Biochrom), supplemented with 15% FBS-fetal bovine serum (Gibco) and 1% penicillin–streptomycin (P/S), as recommended by the suppliers.

#### 2.3.2 HGSOC-AS

HGSOC ascites was collected under sterile conditions during surgery. Cell cultures were generated from 15 mL of fresh ascites seeded in the ratio 1:1 in Dulbecco’s modified Eagle medium-GlutaMAX (Gibco) containing 4.5 g/L glucose, supplemented with 20% FBS and 1% P/S, and incubated at 37 °C and 5% CO_2_. After 3 days, the cells were washed with Hank’s balanced salt solution (HBSS), and fresh media were added. Cells were harvested on early passages (up to three passage stocks).

### 2.4 RNA isolation and quantitative real-time PCR analysis (qRT-PCR)

For the characterization of ovarian cancer samples, we obtained total RNA using approximately 50 mg of fresh frozen tissue, which was first manually homogenized using a pestle and mortar, and then, we isolated RNA from around 3 × 10^5^ cells for therapy response testing.

Total RNA from all sample types was isolated using TRIzol (Invitrogen, Thermo Fisher Scientific), according to the manufacturer’s protocol. The quantity of the RNA was determined by the concentration and purity (A260/A280 and A260/A230), assessed by NanoDrop ND1000 (NanoDrop Technologies, Waltham, MA, United States). Total RNA quality and size distribution were analyzed by chip-based capillary electrophoresis using the Agilent 2100 Bioanalyzer with a 6000 RNA Nano Chip (Agilent Technologies, Santa Clara, CA, United States).

The EM-associated gene expression was quantified by qRT-PCR. cDNA (2000 ng) was synthesized using the High-Capacity cDNA Reverse Transcription Kit (Applied Biosystems, Thermo Fisher Scientific). Sense and antisense primers were designed against published human sequences in Supplementary Table S1. RT-qPCR was performed using the SYBR Green PCR Master Mix (Applied Biosystems, Thermo Fisher Scientific) and 7300 Real-Time PCR System (Thermo Fisher Scientific). The resulting mRNA levels were normalized to the β-actin reference gene. Relative quantification was studied by the 2^−ΔCT^ method (Livak and Schmittgen, 2001; Schmittgen and Livak, 2008).

### 2.5 Immunohistochemistry (IHC)

Cell blocks have been prepared from 3 × 10^6^ AS cells and processed using the ‘cell block’ cytology technique to generate paraffin blocks. The cell block technique has been done using cell suspension materials embedded in paraffin wax, according to the adapted method described in reference (Woods and Stirling, 2019). IHC staining for AS and T was performed on 3-µm-thick section cut from the formalin-fixed cell blocks and paraffin-embedded tumor tissues. Dewaxing and rehydration were completed using the Trilogy heated buffer solution (Cell Marque, Hot Springs, AR), according to the manufacturer’s protocol. In brief, 0.03% hydrogen peroxide (H_2_O_2_) treatment was performed for 10 min, and primary antibodies (CDH1 (Cell Marque; EP700Y; 1:100), CDH2 (Thermo Fisher Scientific; MA-1–91,128; 1:500), KRT18 (Cell Marque; B22.1&B23.1; 1:250), KRT19 (Cell Marque; A-53-B/A2.26; 1:250), and KRT7 (Cell Marque; OV-TL 12/30; 1:250) were incubated overnight at 4°C. The slides were then incubated with peroxidase-labeled polymer conjugated to goat anti-rabbit IgG or goat anti-mouse IgG for 30 min. The sections were stained with DAB and counterstained with hematoxylin. Cells were evaluated with a magnification of ×40. The tissues and AS were evaluated semiquantitatively, assessing the intensity and localization, including membrane, cytoplasm, or nuclear staining.

### 2.6 Functional study and chemotherapy response

#### 2.6.1 Drug treatments

AS-derived cells have been seeded at 1 × 10^4^ cells/well in 96 well-plates with flat-bottoms for 24 h. CIS (Selleckchem, catalog no. S1166, batch no 14) was dissolved in ddH_2_O, and DOX (Selleckchem, Cat no. S1208, Batch no 13) was dissolved in dimethyl sulfoxide (DMSO). Compound effects were measured in a 10-point dilution series after 24 h of incubation. CIS was tested in a range of 800 to 1.562 µM, and DOX, in a range of 25 to 0.048 µM. For CIS experiments, controls consisted of ddH_2_O, and for DOX experiments, controls consisted of DMSO alone (maximal DMSO concentration used was 0.016%). Cell viability was determined with MTT assay (Cell Proliferation Kit, MTT, Roche), and the solubilized formazan product was spectrophotometrically quantified at 570 nm wavelength using the Sunrise Basic Tecan plate reader and Magellan V 6.5 software. Two independent experiments with three technical replicates were conducted for each tested drug. Half-maximal inhibitory concentration (IC_50_) values were calculated with GraphPad Prism 10 software using a dose-response curve fit model by applying the nonlinear log(inhibitor) *versus* response-variable slope (four parameters) equation.

#### 2.6.2 Scratch-healing test

AS cells have been seeded at 1 × 10^5^/well in 12-well culture plates. After 24 h, a straight scratch was carefully made across the cell monolayer using a sterile 10-µL pipette tip to create a wound, and each drug’s median IC_50_ (DOX = 0.6 µM, CIS = 50 µM) was added. Subsequently, the dish was placed under the microscope (IX73 Inverted Microscope, Olympus) to capture images of the scratch at two different time points: immediately after the scratch (0 h) and 24 h later (24 h). These images were analyzed using ImageJ software. The extent of wound closure was measured by quantifying the reduction in scratch width over time.

#### 2.6.3 Apoptosis assay

Cells have been lysed with Milliplex Map Lysis Buffer 1× (Merck Millipore), supplemented with protease inhibitors and assayed using ProcartaPlex multiplex immunoassay (Thermo Fisher Scientific), according to the manufacturer’s instructions. All samples were measured in duplicate. Data were initially acquired as mean fluorescence intensity (MIF), and the ratio of fluorescence to standard magnetic microspheres was then calculated. A series of calibrators were analyzed, and standard curves and concentrations were obtained using Bio-Plex Manager Software.

#### 2.6.4 Western blotting

AS cells were lysed in RIPA buffer (MILLIPLEX MAP Lysis Buffer 1×, Merck Millipore) with the protease inhibitor (Protease Inhibitor Cocktail 50×, Promega). A measure of 30 μg of proteins were electrophoresed on a 15% and 8% SDS-polyacrylamide gel and then transferred to a polyvinylidene difluoride (PVDF) membrane (Bio-Rad Laboratories). After the blocking step with 5% nonfat dried milk (PanReac AppliChem) diluted in PBST (1× phosphate-buffered saline with Tween 20) for 2 h at room temperature, the primary antibodies mouse anti-KRT19 (A53-B/A2.26-Ks19.1 Thermo Fisher), rabbit anti-CDH2 (D4R14, Cell Signaling), and HRP-GAPDH (PA1-987 HRP, Invitrogen) were incubated overnight at 4°C. Afterward, the membrane was washed three times with PBST and incubated with goat anti-rabbit (G21234, Invitrogen) and m-IgGk BP-HRP (SC-516102, Santa Cruz) for 1 h at room temperature and washed again in PBST buffer. The signals were detected using Pierce ECL Western blotting Substrate (Thermo Fisher Scientific) and OPTIMAX X-ray film processor (Protec). The densitometric analysis quantified protein expression by ImageJ Software.

### 2.7 Statistical analysis

Quantitative and semiquantitative analyses for IHC tissue markers were performed with the support of experienced pathologists (VH).

In descriptive statistics, data are presented as n (%) or median (interquartile range (IQR): Q1 and Q3). Statistical significance of univariate analysis was determined by the Mann–Whitney–Wilcoxon test with *p*-values calculated by the exact method and the Kruskal–Wallis test for ordinal or continuous variables with a non-normal distribution. For normal distribution (evaluated with the Shapiro test), we used an unpaired *t*-test with Welch’s correction and one-way analysis of variance (ANOVA) and a chi-squared test for dichotomous variables, followed by the Bonferroni *post hoc* test for multiple comparisons. All *p*-values were based on two-sided hypothesis tests, and p <0.05 was considered statistically significant.

Wilcoxon tests were used for comparisons between two independent groups. AS results were presented as the means of two independent experiments ± SEM, and the comparisons between two groups (sensitive vs resistant) were performed using Mann–Whitney t-tests. Differences were considered statistically significant at *p* < 0.05.

We used log-transformed TPM values to normalize the mRNA expression of EM-associated markers to obtain a normal distribution of data for all RNA-seq transcriptomic results.

A Cox proportional hazards regression was used for univariate and multivariate analyses of prognostic variables for overall survival. Multivariate survival analysis was performed for all variables that indicated significant diagnostics, based on the scaled Schoenfeld residuals, to identify independent predictors of survival. The hazard ratio (HR) and the corresponding 95% confidence interval (95% CI) were calculated. Overall survival was defined as the interval between the date of surgery and the date of death or the end of follow-ups. The R packages used in the survival analysis are gtsummary 1.7.2, survival 3.5.7, survminer 0.4.9, and flextable 0.9.4. The statistical analysis used GraphPad Prism 10 for Windows (GraphPad Software, Inc.) and R 4.3.0 software.

**FIGURE 1:**
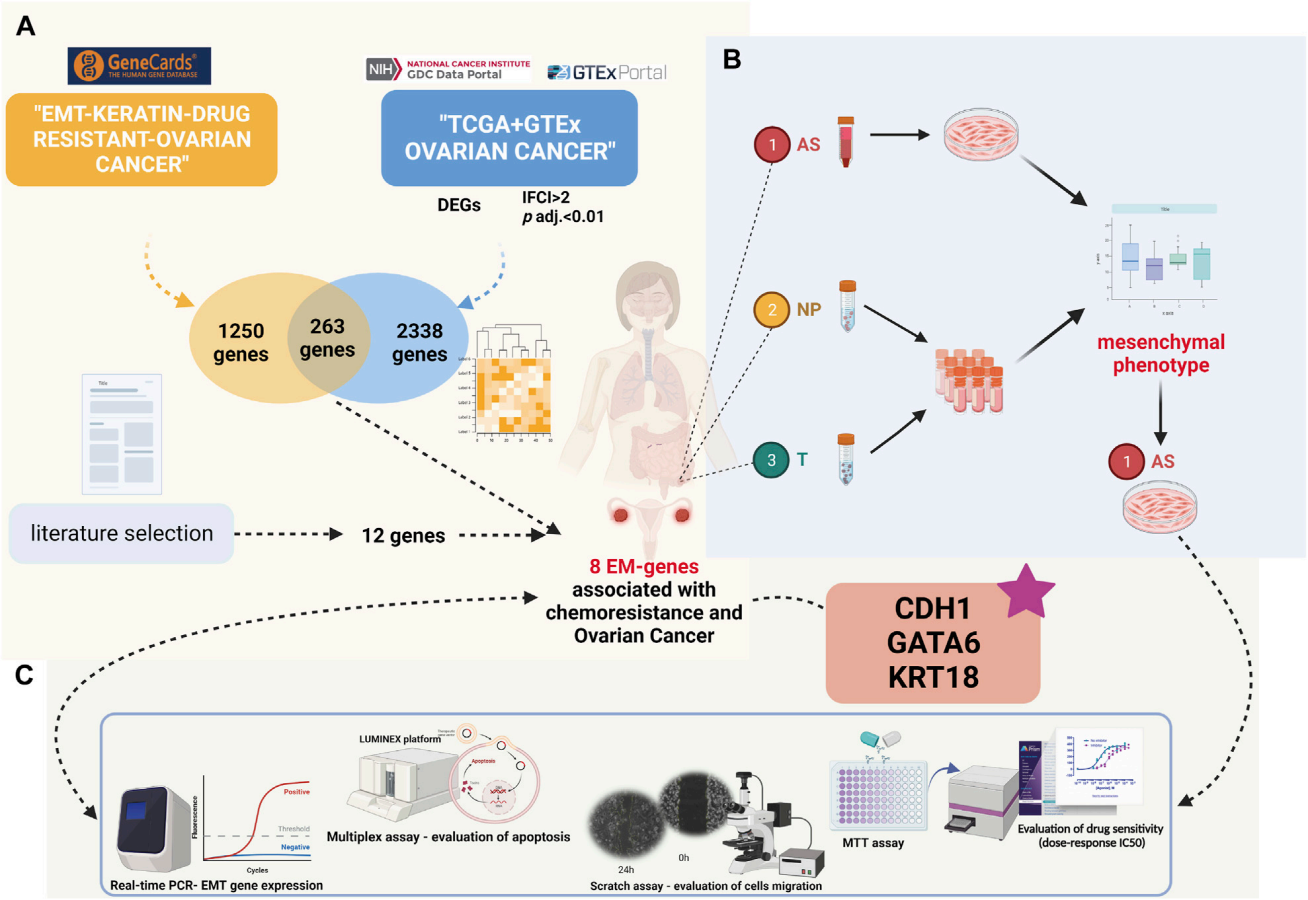
Present study’s design (created with biorender.com accessed on December 2023): **(A)** Differential gene expression and Gene Ontology analyses in the TCGA-OV cohort compared to GTEx ovarian normal tissue. Intersection of significantly expressed genes with EM-associated genes related to chemoresistance from the GeneCards database. **(B)** Patient sample inclusion and HGSOC ascites primary culture establishment. **(C)** Comprehensive analysis of HGSOC ascites primary cultures at functional and molecular levels.

Figure 1 presents the workflow strategy of this study.

## 3 Results

### 3.1 Investigation of EM markers related to chemoresistance

**FIGURE 2:**
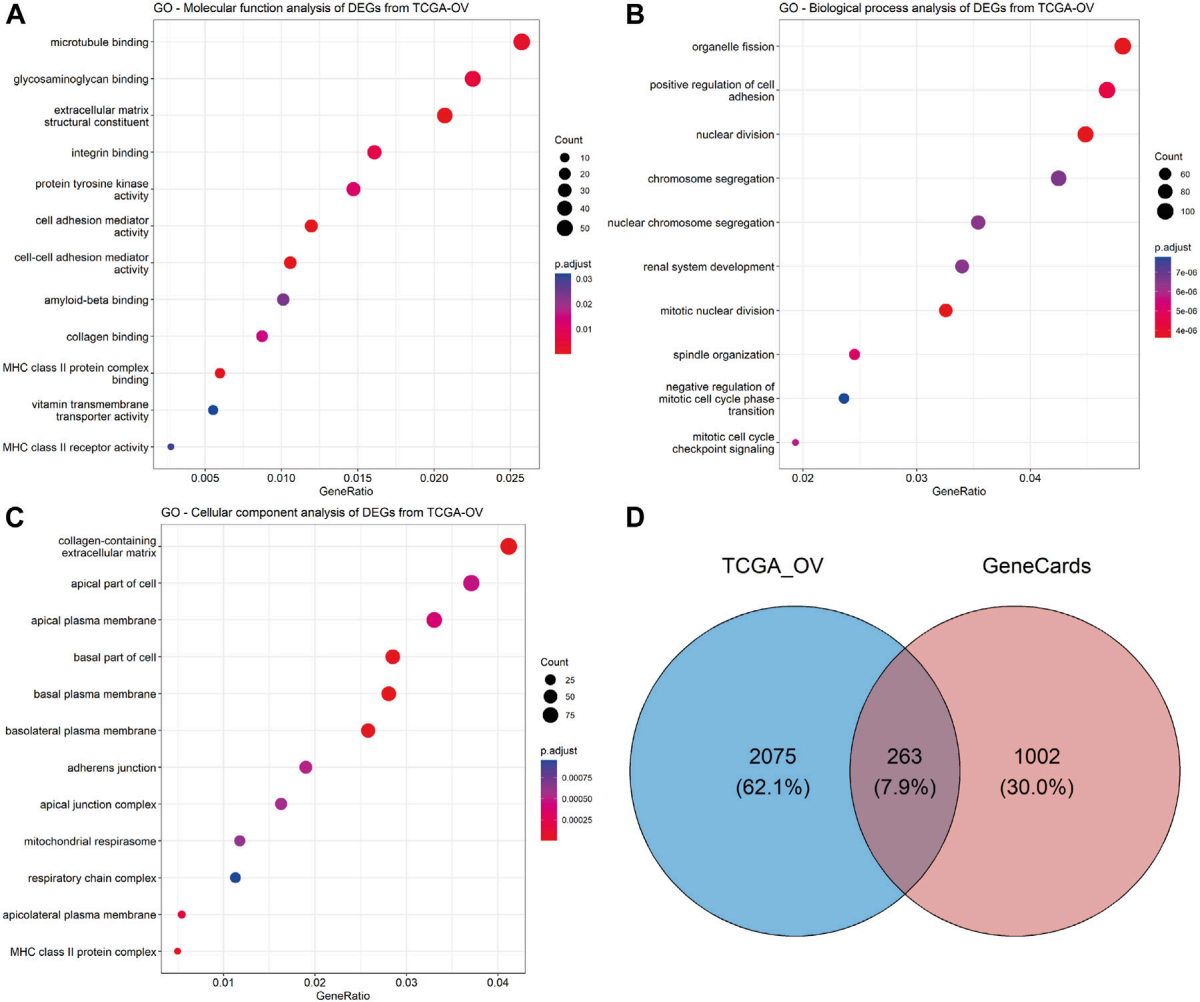
Exploration of the predictive biomarkers related to chemoresistance in HGSOC patients. GO enrichment analysis of significant DEGs from TCGA-OV datasets was performed using the clusterProfiler (version 4.8.2) R package: **(A)** molecular function, **(B)** biological process, and **(C)** cellular component analyses. The dot size represents the count of relative genes, and the gradient color represents the adjusted *p*-value. **(D)** Venn diagram for the intersection of significant DEGs from TCGA-OV and GeneCards databases.

To explore the predictive biomarkers related to chemoresistance in HGSOC, we first performed *in silico* analysis. First, we obtained 1,278 upregulated genes and 1,060 downregulated genes in OV compared to normal ovarian tissues using TCGA and GTEx cohorts (Supplementary Table S2). Gene Ontology (GO) analysis found that cell–cell adhesion molecular functions (Figure 2A; Supplementary Table S3) and the positive regulation of cell–cell adhesion biological function were among the top enriched (Figure 2B; Supplementary Table S4). Notably, extracellular matrix and cellular adhesion were the top 10 ranked cellular components determined by GO analysis (Figure 2C; Supplementary Table S5). The most important molecular functions related to these genes are EMT and KRTs, defined as EM-associated genes.

After the intersection of TCGA-significant DEGs and EM-associated genes related to chemoresistance from the GeneCards database (Supplementary Table S6), we obtained 263 genes (Figure 2D; Supplementary Table S7). A total of eight genes (*CDH1*, *CDH2*, *VIM*, *GATA6*, *EPCAM*, *KRT7*, *KRT18*, and *KRT19*) were correlated with chemoresistance and HGSOC after relevant literature investigation (Supplementary Table S8) and were included in further analysis.

### 3.2 Generation of primary cultures from HGSOC-AS

The 12 patients included in this study had a median age of 59 (interquartile range 54–65 years old). All patients recruited were diagnosed with HGSOC. A total of 67% of patients were in stage IIIC. The median overall survival in this cohort was 23 months. Clinical and pathologic parameters are summarized in Table 1.

**TABLE 1:** Demographic and clinical–pathological data on HGSOC patients included in the prospective study.

Characteristic	N	N = 12^a^
Age (years)	12	
Median (IQR)		59 (54, 65)
Differentiation degree	12	
G1		1 (8.3%)
G2		1 (8.3%)
G2–G3		2 (17%)
G3		8 (67%)
Staging (FIGO)	12	
IIA		1 (8.3%)
IIIA2		1 (8.3%)
IIIB		1 (8.3%)
IIIC		8 (67%)
IVB		1 (8.3%)
Tumor size (cm)	12	
Median (IQR)		4.3 (2.5, 7.3)
Overall survival (months)	12	
Median (IQR)		23 (11, 34)
CA125 (ng/mL)	11	
Median (IQR)		445 (392, 807)
CA15-3 (ng/mL)	10	
Median (IQR)		58 (23, 125)
CEA (ng/mL)	10	
Median (IQR)		1.41 (0.60, 1.86)
CA19-9 (U/mL)	10	
Median (IQR)		14 (4, 52)

^a^ Median (IQR), percentage (%).

**FIGURE 3:**
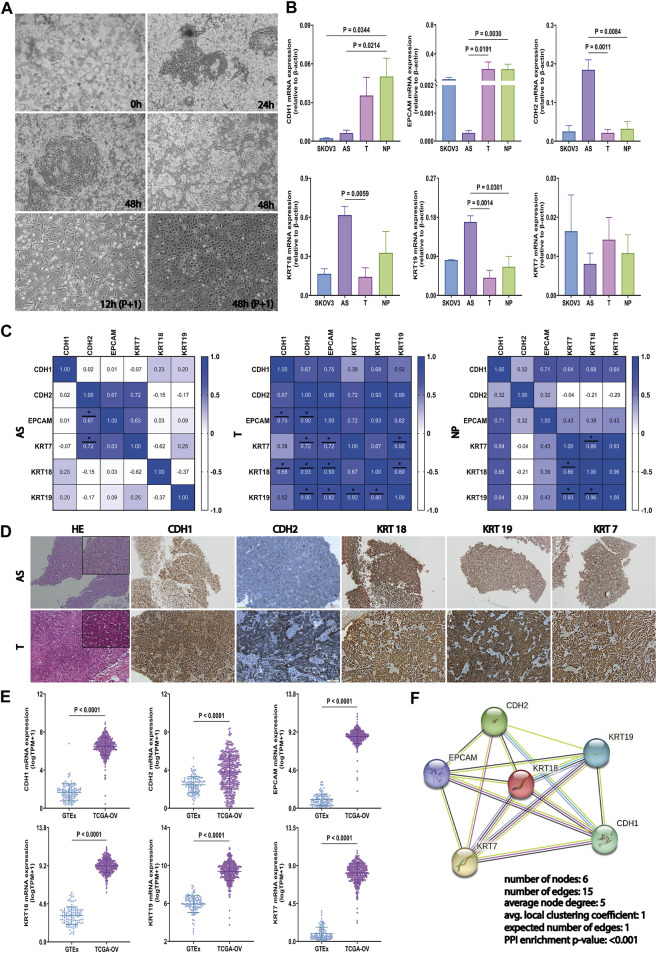
Generation of primary cultures from HGSOC-AS and investigation of EM-associated markers in ascites, tumors, and peritoneal nodule. **(A)** Representative HGSOC ascites-derived primary cultures with the presence of a significant amount of spheroids in culture, immediately after cell isolation (0h, left-upper corner), clusters from which tumor epithelial cells migrate 24 h after seeding (24 h-right-upper corner), and clusters from which tumor epithelial cells migrate 48 h after seeding (48 h-middle). In the first passage, after 12 h (P+1), the tumoral cells presented an epithelial morphology, and after 48 h (P+1), the cells reached confluence. **(B)** *CDH1*, *EPCAM*, *CDH2*, *KRT18*, *KRT19*, and *KRT7* gene expression in SKOV3, AS (n = 9), T (n = 9), and NP (n = 7) detected by qRT-PCR. Data are represented as mean ± SEM. **(C)** Correlation matrix for *CDH1*, *EPCAM*, *CDH2*, *KRT18*, *KRT19*, and *KRT7* gene expression in each tumor sample derived from HGSOC patients: AS (left), T (middle), and NP (right) (two-sided Spearman’s correlation test and gradient color bar represent Spearman correlation coefficients). **(D)** Representative immunostaining images illustrating EM-associated marker protein expression (*CDH1*, *CDH2*, *KRT18*, *KRT19*, and *KRT7*) and hematoxylin–eosin staining in AS (upper panel) and corresponding T (lower panel) (scale bar, 100 μm). **(E)** Investigation of the EM-associated gene expression profile in the tumor ovarian TCGA cohort (n = 421) compared with normal ovarian GTEx datasets (n = 108). mRNA expression levels of EM-associated markers were normalized by log_2_ (TPM+1). **(F)** The PPI analysis among EM-associated markers was acquired using the STRING (https://www.string-db.org/) web-tool database. All *p* < 0.05 is considered statistically significant.

To provide relevant molecular aspects about the HGSOC microenvironment, we established 12 HGSOC ascites-derived primary cultures, as described elsewhere (Shepherd et al., 2006; Theriault et al., 2013). Their malignancy has been confirmed through cytological detection in the Pathological Anatomy Laboratory of the Fundeni Clinical Institute. Thus, rich cell sediment was observed, represented by tumor cells, red blood cells, frequent inflammatory cellular elements, and enlarged cells isolated and grouped together, some with three-dimensional uneven appearance and enlarged slightly uneven nuclei, which are often eccentric and tachromic; the cytoplasm was basophilic with vacuolations. Immediately after isolation (0 h), ascites-derived cells revealed wide heterogeneity, including suspended cell aggregates called spheroids. After 24 h (24 h), the presence of spheroids in suspension was still detectable, but adherent cells became predominant. After first passage, at 48 hours, 48 h (P+1), the confluent AS cell monolayer illustrates a “cobblestone” phenotype. The pattern of *in vitro* proliferation analysis shows variations, with primary cells derived from ascites reaching confluence in 6–10 days, depending on the biological variability of each patient (Figure 3A). AS, T, and NP samples of HGSOC patients were followed by subsequent mRNA expression analyses and *in vitro* functional tests.

### 3.3 Evaluation of EM-associated markers in ascites, tumors, and peritoneal nodule

To explore the epithelial–mesenchymal phenotype of AS, T, and NP, in our cohort and SKOV3 cell line, the expression of the most used EM-associated genes (CDH1, CDH2, EPCAM, KRT7, KRT18, and KRT19) has been evaluated by qRT-PCR. The AS has a significantly lower level of CDH1 compared to NP (*p* = 0.0214), the same as SKOV3 to NP (*p* = 0.0344). The EPCAM downregulation was detected in AS cells but not in SKOV3, with *p* = 0.0191 compared to T and *p* = 0.0030 compared to NP.

On the other hand, a significantly high mRNA level of CDH2 was observed in AS, compared to T (*p* = 0.0011) and NP (*p* = 0.0084). The expression of KRT18 was considerably higher in AS compared to T (*p* = 0.0059), which is in convergence with KRT19 in AS with *p* = 0.0014 compared to T and *p* = 0.0301 compared to NP. For KRT7, no significant differences were noticed between the four types of samples derived from HGSOC (Figure 3B).

Spearman’s rank correlation showed that there was a strong positive relation between the levels of CDH2—KRT7 and CDH2—EPCAM in AS and T, respectively, with r > 0.6 and *p* < 0.05. Moreover, all investigated markers, except KRT7–CDH1, KRT19–CDH1, and KRT7–KRT19, have a significant positive correlation in T (r > 0.6, *p* < 0.05). In contrast, only KRT markers exhibit a strong positive correlation in NP (r > 0.6, *p* < 0.05) (Figure 3C). The distinct patterns of association of each EM associated marker with cytokeratin demonstrate the unique expression profile of each type of biological material derived from HGSOC, highlighting that AS has the greatest heterogeneity and a mesenchymal phenotype.

Therefore, we also validated the results of marker expressions by IHC in tumor tissues and AS, respectively. The representative images for CDH1, CDH2, KRT18, KRT19, and KRT7 are shown in Figure 3D, and the IHC scores are detailed in Supplementary Table S9.

In addition to these, we analyzed TCGA-OV and GTEx against our list of EM-associated genes. Figure 3E shows that the EM-associated markers were significantly upregulated in OV tissues compared to normal ovarian tissues.

Furthermore, we acquired and visualized the protein–protein functional associations via the STRING database (https://www.string-db.org/) (von Mering et al., 2003). Therefore, we validated the interaction between the EMT and KRT markers (PPI enrichment *p*-value <0.001) (Figure 3F). Results show that AS presents a mesenchymal phenotype that enhances the aggressivity of HGSOC. Thus, this model is used for further analysis to predict drug response.

### 3.4 The establishment of chemosensitivity in the AS model

#### 3.4.1 Determination of drug response in the AS model

To investigate the effect of drug response on AS, we have analyzed two important HGSOC drugs that induce apoptosis by different mechanisms (Morgan et al., 2013) because cisplatin represses cell division and tumor growth by interfering with DNA replication and causing DNA cross-linking (Siddik, 2003; Helm and States, 2009; Makovec, 2019), while doxorubicin intercalates with DNA, inhibits topoisomerase II, and promotes reactive species accumulation (Thorn et al., 2011; Henri et al., 2023).

**FIGURE 4:**
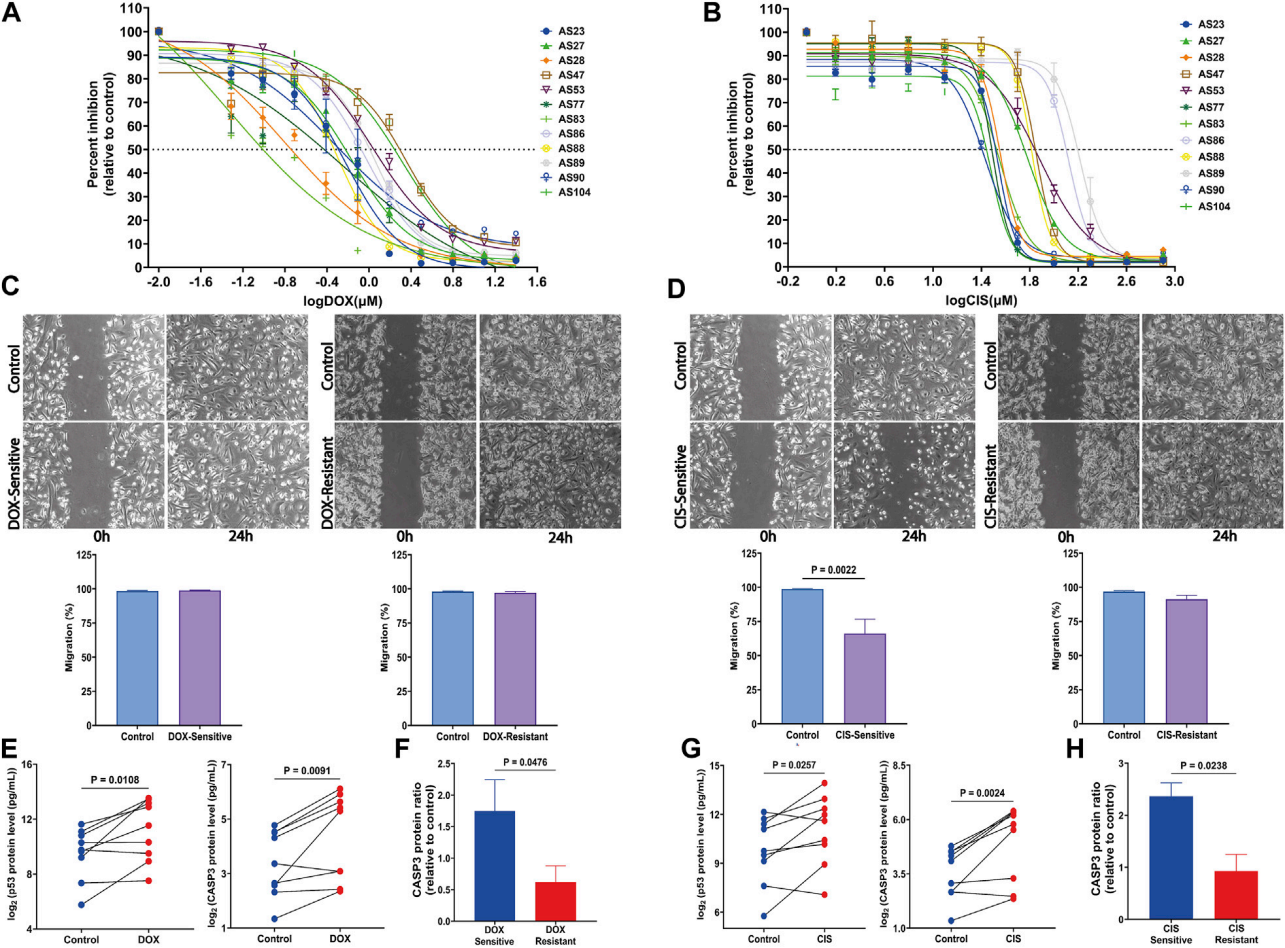
Response of HGSOC AS primary cells to tested drugs and the assessment of apoptosis markers changes. **(A)** Sensitivity dose curves for 12 AS primary cells from HGSOC patients against two drugs DOX and **(B)** CIS. The dashed line mark indicates 50% inhibition by the drugs. **(C)** Representative images of wound-healing tests of AS primary cells exposed to DOX and **(D)** CIS for 24 h. The relative wound closure was indicated as migration (%) (n = 4 AS with three independent experiments each; scale bar, 200 μm). **(E,F)** p53 and CASP3 protein levels were measured by multiplex apoptosis assay in AS untreated and treated with DOX and **(G,H)** CIS (n = 9). Data are represented as mean ± SEM. All *p* < 0.05 is considered statistically significant.

First, we detected the cell viability using an MTT assay on 12 AS. Both drugs’ working concentrations (0.04875–25 µM DOX and 1.562–800 µM CIS) significantly decreased viability in all cultures tested in a dose-dependent manner (Figures 4A, B). Furthermore, we established the IC_50_ value after 24 h drug exposure. Thus, we used each drug’s median IC_50_ (DOX = 0.6 µM, CIS = 50 µM) for AS cultures in subsequent analyses.

In addition, for AS, the IC_50_ value of drugs equal to or less than their respective median IC_50_ was considered a sensitive culture, while IC_50_ values greater than the median IC_50_ values were regarded as a resistant culture.

Therefore, cell migration investigated using the scratch assay demonstrates a significant decrease in the CIS-sensitive group compared to its untreated control (*p* = 0.0022) (Figure 4D). In contrast, migration is not affected by DOX, either in resistant or sensitive groups (Figure 4C).

#### 3.4.2 CASP3 expression is positively correlated with AS sensitivity to cisplatin and doxorubicin

To determine drug treatment resistance, apoptosis markers, caspase-3 (CASP3), and tumor protein P53 (p53) were measured using a multiplex apoptosis assay and the Luminex platform. As shown in Figure 4E, CASP3 and p53 significantly increased after DOX (*p* = 0.0091 and *p* = 0.0108, respectively) and CIS (*p* = 0.0024 and *p* = 0.0238, respectively) treatment (Figure 4G). Interestingly CASP3 levels were elevated in both sensitivity-drug groups (Figures 4F, H). These findings suggest that DOX and CIS repress proliferation and promote apoptosis by increasing CASP3 and p53 in the sensitive-AS group. Furthermore, CIS suppresses migration, while DOX does not affect it.

### 3.5 Drug treatment regulates EM-associated markers in AS

**FIGURE 5:**
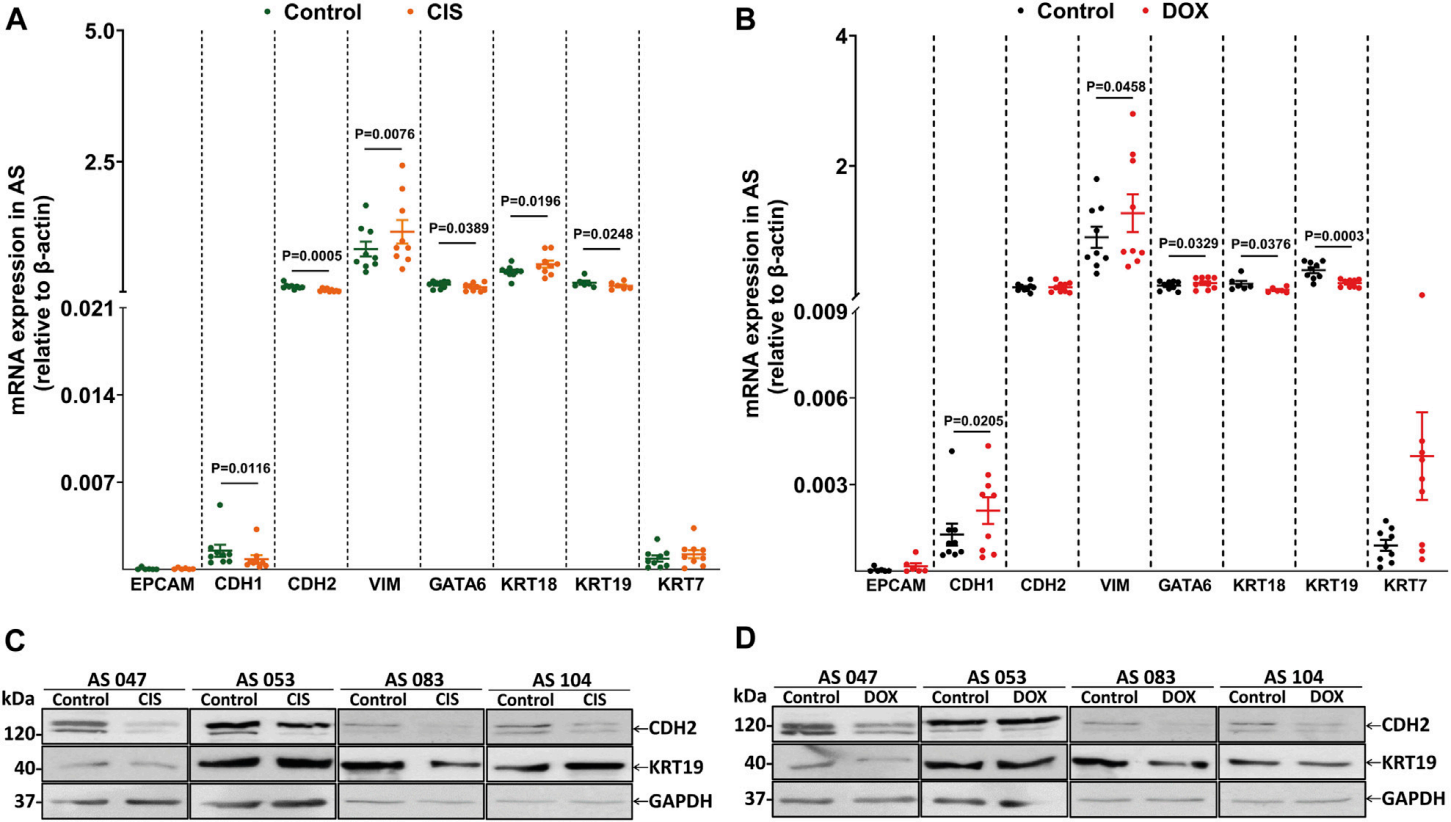
Drug treatment regulates EM-associated gene expression in AS primary cultures. **(A)** Evaluation of eight EM-associated gene profiles (*EPCAM*, *CDH1*, *CDH2*, *VIM*, *GATA6*, *KRT18*, *KRT19*, and *KRT7*) related to CIS and **(B)** DOX responses in AS primary cultures (n = 9). All *p* < 0.05 is considered statistically significant. **(C)** Western blotting showing CDH2 and KRT19 protein levels in AS treated with CIS and **(D)** DOX compared to untreated cultures. GAPDH was used as the loading control (n = 4).

In order to explore the correlation of EM-associated markers and DOX and CIS ascites culture treatment, we have evaluated the mRNA expression by qRT-PCR. The results showed that VIM (*p* = 0.0076) and KRT18 (*p* = 0.0196) were significantly upregulated in CIS-treated AS compared to untreated-AS (control), while CDH1 (*p* = 0.0116), CDH2 (*p* = 0.0005), KRT19 (*p* = 0.0248), and GATA6 (*p* = 0.0389) were decreased (Figure 5A). In addition, upon treatment with DOX, the expression of CDH1 (*p* = 0.0205), VIM (*p* = 0.0458), and GATA6 (*p* = 0.0329) was increased, and the expression of KRT18 (*p* = 0.0376) and KRT19 (*p* = 0.0003) was downregulated (Figure 5B). CDH2 and KRT19 expression has been assessed by Western blotting, indicating a decreased level after CIS/DOX treatment (Figure 5 C, D).

Therefore, these data demonstrate that CIS treatment induces mesenchymal phenotype by VIM expression; this mechanism has been previously associated with chemoresistance (Li et al., 2011; Sun et al., 2012; Li et al., 2015). Interestingly, only DOX increases CDH1 significantly, possibly inducing an invasive intermediate-EMT state (Klymenko et al., 2017).

### 3.6 Different chemotherapies modulate EM-associated markers in HGSOC cell cultures and patients

We have validated the mRNA expression changes of EM-associated genes in drug-resistant and drug-sensitive cells, as well as in patients.

**FIGURE 6:**
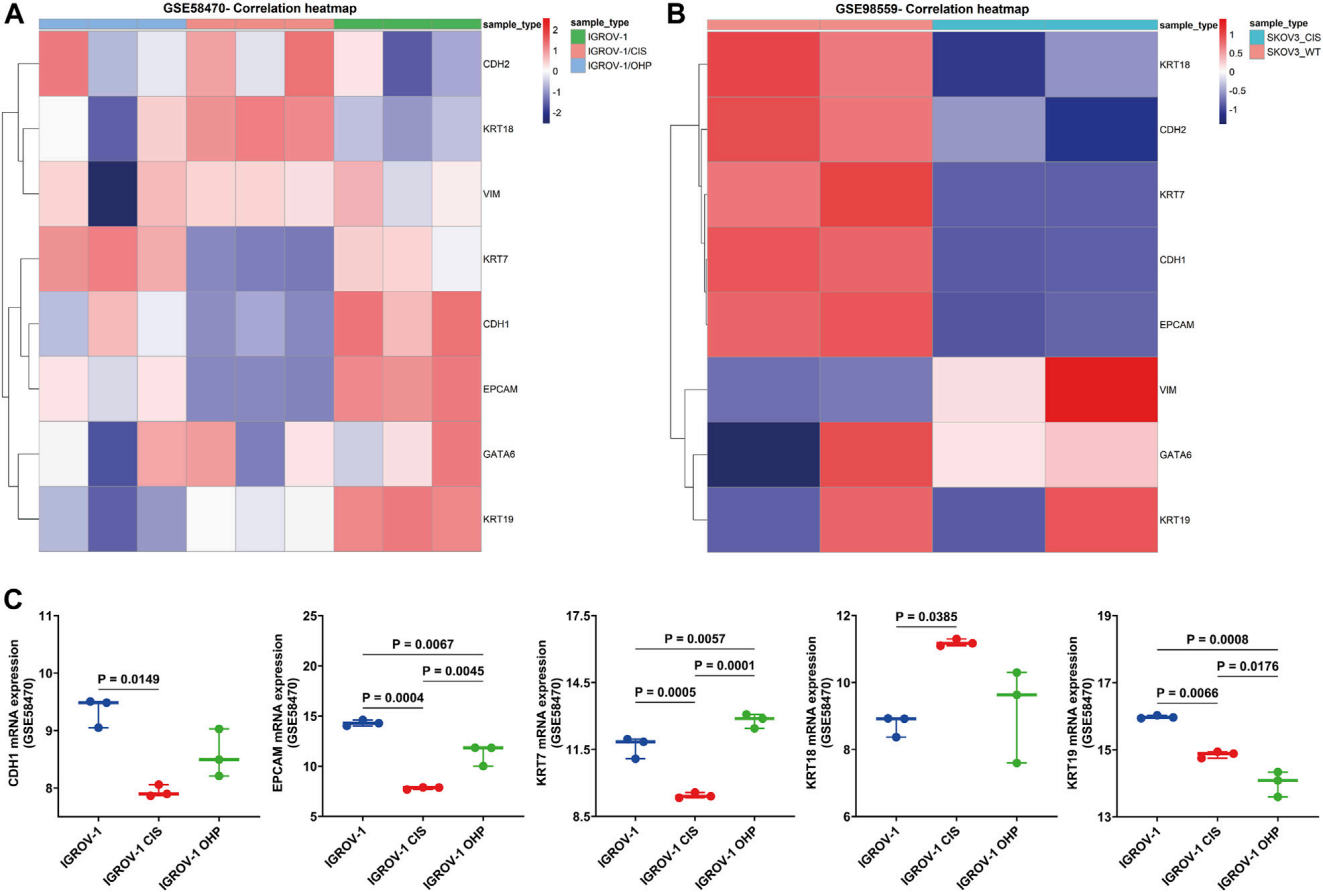
Validation of the EM-associated gene expression profile in platinum-resistant HGSOC cell cultures. **(A)** Heatmap correlation of the EM-associated gene expression profile in parental cisplatin-sensitive IGROV-1, the oxaliplatin-resistant IGROV-1 OHP, and cisplatin-resistant IGROV-1 CIS cell lines generated by the exposure of parental cells to OHP and, respectively, to CIS (GSE58470). Patterns of mRNA transcript abundance were significantly changed in IGROV-1 CIS compared to IGROV-1. **(B)** Heatmap correlation of the EM-associated gene expression profile in SKOV3 wild-type cells and SKOV3 CIS-resistant cells (GSE98559). Similarly, EM-associated genes indicated significant transcriptomic profile changes in SKOV3 CIS-resistant cells. **(C)** Investigation of the EM-associated gene expression profile in GSE58470 cell lines (n = 3 with three independent experiments each) indicated that CDH1, EPCAM, and KRT7 were significantly decreased and KRT18 is increased in IGROV-1 CIS, compared to IGROV-1 cell lines. All *p* < 0.05 is considered statistically significant.

First, we investigated GSE58470, which includes IGROV-1 (parental cell, an established cell line to study chemoresistance in ovarian cancer properly) and two platinum-resistant variants (IGROV-1 CIS and IGROV-1 OHP) (Arrighetti et al., 2016). The heatmap in Figure 6A shows the correlation of EM-markers in platinum-resistant cells compared to IGROV-1. On one hand, CDH1, EPCAM, KRT7, and KRT19 were significantly lower in platinum-resistant variants than in parental cells (*p* < 0.05); on the other hand, KRT18 was upregulated in IGROV-1 CIS (*p* = 0.0385), while KRT7 increases in IGROV-1 OHP compared to IGROV-1 (*p* = 0.0057) and IGROV-1 CIS (*p* = 0.0001) (Figure 6C).

Furthermore, we analyzed the expression of EM-associated markers in SKOV3 CIS-resistant and wild-type SKOV3 cell lines using GSE98559 (Meng et al., 2018; Zhou et al., 2018). Epithelial markers (KRT18, KRT7, CDH1, and EPCAM) were alleviated in SKOV3 CIS, compared to wild-type SKOV3, while mesenchymal markers (VIM) were highly expressed, except CDH2, which decreased after CIS exposure (Figure 6B).

**FIGURE 7:**
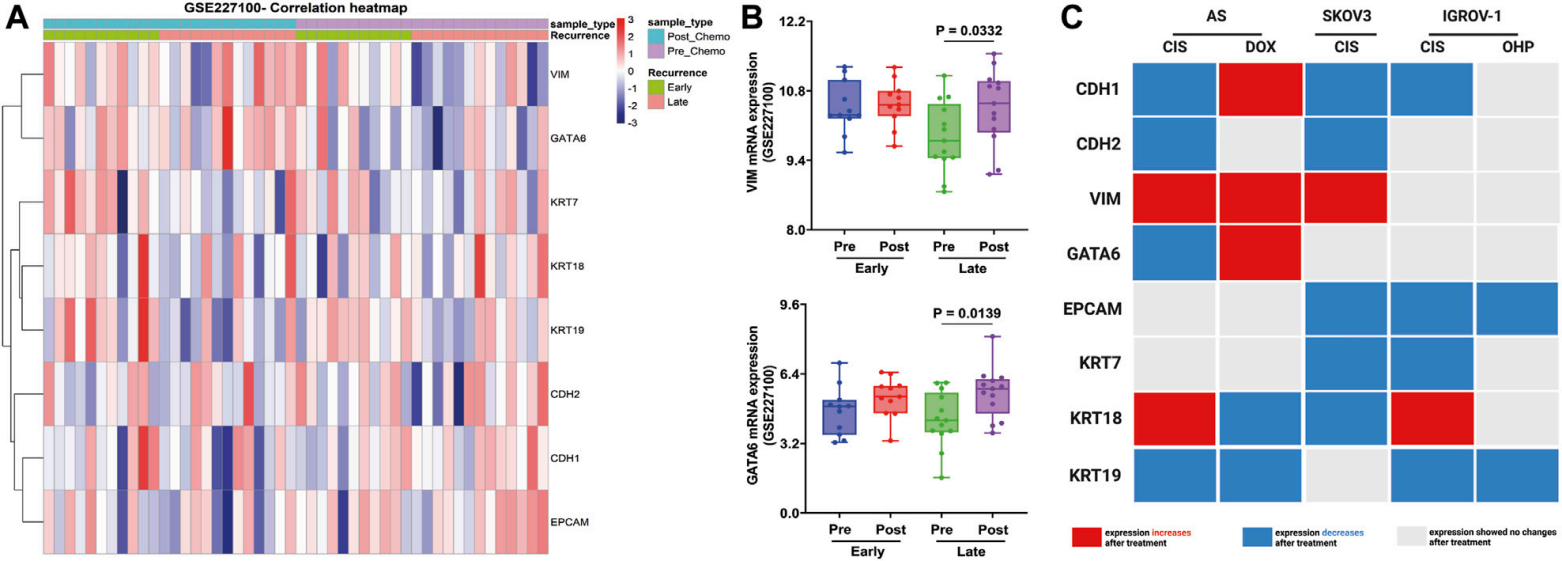
Validation of EM-associated gene expression changes in tumor samples obtained from HGSOC patients treated with combination chemotherapy and associated with recurrence status. **(A)** Heatmap correlation of the EM-associated gene expression profile in 24 tumor samples before and after treatment with six cycles of CARB and PAX (GSE227100). Each column indicates distinct tumor samples. **(B)** Ovarian cancer patients with late recurrence indicated a significantly upregulated GATA6 and VIM mRNA expression after combination chemotherapy. All *p* < 0.05 is considered statistically significant. **(C)** Signature of EM-associated gene expression in synergy with chemotherapy in HGSOC AS primary cultures and cell lines (red box represents increased mRNA expression, blue box represents decreased mRNA expression, and gray represents without mRNA expression changes after treatment).

Finally, these EM-associated markers were investigated in patient samples from GSE227100 (Figure 7A), which contained 24 HGSOC patients before and after they were treated with six cycles of CARB and PAX combination chemotherapy (pre-C/T; post-C/T) (Adzibolosu et al., 2023). The results showed that the expression of VIM and GATA6 increased significantly after chemotherapies in the late recurrence group (Figure 7B).

These findings show that mRNA changes could differentiate between administrated treatments. Indeed, CIS thereby attenuates CDH1 and KRT19 expressions in all cells treated. In contrast, DOX contributes to the induction of the intermediate-EMT state by the expression of CDH1, VIM, and GATA6 (Figure 7C).

### 3.7 Clinical predictive value of EM-associated markers in HGSOC patients

To evaluate the clinical prognosis of the eight EM-associated genes, we have performed uni- and multivariate Cox regression analysis in OV patients from TCGA cohort. The univariate Cox proportional hazards regression analysis confirmed the higher risk of death for patients with a more advanced stage, stage IV (HR = 2.14, *p* = 0.047), high mRNA expression of the KRT7 marker (HR = 1.37, *p* = 0.012), and with the elevated mRNA expression of the KRT19 marker (HR = 1.33, *p* = 0.022). In addition, primary therapy outcome status in correlation with overall survival in univariate Cox regression indicated a lower risk of death for patients with stable disease (HR = 0.49, *p* = 0.036), complete remission/response (HR = 0.17, *p* < 0.001) compared to the progressive disease group, and high mRNA expression of EPCAM (HR = 0.73, *p* = 0.011) (Table 2). No association was detected between overall survival and the remaining EM-associated markers.

**TABLE 2:** Univariate and multivariate Cox proportional hazards regression analyses of EM-associated gene expression markers in correlation with the overall survival of HGSOC patients (TCGA).

	Summary data	Univariate				Multivariate			
Characteristic	N = 420^a^	N	HR^b^	95% CI^b^	*p*-value	N	HR^b^	95% CI^b^	*p*-value
Tumor stage		417				335			
Stage I/II	25 (6.0%)		—	—			—	—	
Stage III	328 (79%)		1.73	0.85, 3.51	0.13		2.33	0.92, 5.87	0.073
Stage IV	64 (15%)		2.14	1.01, 4.53	0.047		1.82	0.69, 4.82	0.2
Primary therapy outcome		337				335			
Progressive disease	32 (9.5%)		—	—			—	—	
Partial remission/response	47 (14%)		0.72	0.44, 1.16	0.17		0.63	0.38, 1.05	0.075
Stable disease	23 (6.8%)		0.49	0.25, 0.96	0.036		0.46	0.23, 0.93	0.031
Complete remission/response	235 (70%)		0.17	0.11, 0.26	<0.001		0.16	0.10, 0.25	<0.001
CDH1 mRNA expression		420				335			
Low	210 (50%)		—	—			—	—	
High	210 (50%)		1.09	0.85, 1.39	0.49		1.13	0.83, 1.54	0.4
CDH2 mRNA expression		420				335			
Low	210 (50%)		—	—			—	—	
High	210 (50%)		0.85	0.67, 1.08	0.19		0.76	0.57, 1.02	0.071
KRT7 mRNA expression		420							
Low	210 (50%)		—	—					
High	210 (50%)		1.37	1.07, 1.75	0.012				
KRT18 mRNA expression		420				335			
Low	210 (50%)		—	—			—	—	
High	210 (50%)		1.08	0.85, 1.38	0.52		1.08	0.81, 1.45	0.6
KRT19 mRNA expression		420				335			
Low	210 (50%)		—	—			—	—	
High	210 (50%)		1.33	1.04, 1.70	0.022		1.37	1.02, 1.84	0.035
EPCAM mRNA expression		420				335			
Low	210 (50%)		—	—			—	—	
High	210 (50%)		0.73	0.57, 0.93	0.011		0.78	0.57, 1.05	0.10
VIM mRNA expression		420				335			
Low	210 (50%)		—	—			—	—	
High	210 (50%)		0.96	0.75, 1.22	0.73		0.89	0.66, 1.19	0.4
GATA6 mRNA expression		420				335			
Low	210 (50%)		—	—			—	—	
High	210 (50%)		1.11	0.87, 1.42	0.40		1.07	0.80, 1.44	0.6

^a^ Percentage (%).

^b^ HR, hazard ratio; C, confidence interval.

Finally, we investigated the significant variables to describe how they correlate with overall survival. To this end, we performed a multivariate Cox regression analysis using the proportional hazards assumption for the Cox model using statistical tests and graphical diagnostics based on the scaled Schoenfeld residuals, including all variables (Supplementary Figure S3).

The multivariate Cox proportional hazards regression analysis shows that a higher risk of death is directly associated with the high mRNA expression of the KRT19 marker (HR = 1.43, *p* = 0.018) and that a lower risk of death is correlated with stable disease (HR = 0.45, *p* = 0.028) and complete remission/response (HR = 0.15, *p* < 0.001) compared to the progressive disease group (Table 2). After controlling for the confounding factors, multivariate Cox regression analysis reveals that primary therapy outcomes and mRNA expression of the KRT19 marker are independent variables of overall survival.

## 4 Discussion

Growing evidence has suggested that overexpression of EMT and KRT markers is positively correlated with the progression and occurrence of various malignant carcinomas, including HGSOC (Xiao and He, 2010; Wang and Zhou, 2011; Rosso et al., 2017; Brabletz et al., 2018; Loret et al., 2019; Zhang et al., 2020; Sohn et al., 2021; Ghionescu et al., 2023; Lu et al., 2023; Machino et al., 2023). Numerous studies have been conducted to establish the EMT index and its association with patient clinicopathologic features, aiming to improve the patient prognosis. Sohn HS et al. developed an EMT-TF-based prognostic index for patients by whole-exome and RNA sequencing. According to the study, the mesenchymal type, characterized by the activation of EMT-TFs and less genomic modification, is more aggressive than the homologous recombination repair (HRR)-activated type with deficiencies in HRR genes (Sohn et al., 2021). Using single-cell sequencing, Xu et al. found that the expression of NOTCH receptor 1 (NOTCH1), SNAI2, transforming growth factor beta receptor 1 (TGFBR1), and Wnt family member 11 (WNT11) EMT-associated genes is correlated with poor patient survival. In addition, primary matrix cancer-associated fibroblasts (mCAFs) can promote EMT and cell invasion by interacting with tumoral and immune cells (Xu et al., 2022). In addition, a recent study has revealed the correlation between N6-methyladenosine (m6A) modification regulators and EMT markers in OV development (Zhang et al., 2023).

However, no independent analysis of the predictive markers related to EM-associated genes and chemoresistance in HGSOC ascites has been conducted. This study found a new gene signature to guide therapy in this pathology, based on ascites, the most accessible sample. First, as a result of our analyses, the most relevant molecules from EMT and KRT families have been investigated in all sample types that could be collected from HGSOC patients (primary tumor, metastatic peritoneal nodules, and tumor ascitic fluids) and in SKOV3, an intermediate mesenchymal ovarian cell line (Rosso et al., 2017). These data show the first EM-marker analysis in the most relevant types of samples in HGSOC and indicate that ascites has a mesenchymal phenotype. In contrast, T and NP have epithelial phenotypes and similarities in gene expression. Li Y et al. have reported that CDH1 decreases in ascites, while CDH2, VIM, and KRT-19 increase in AS, compared to tumoral tissues (Rizvi et al., 2013; Li et al., 2021), so our data conform to the previous reports.

As discussed above, the heterogeneity of HGSOC and ascites presence affects therapy efficacy (Pogge von Strandmann et al., 2017; Zhang et al., 2018). Many studies have used cell-free ascites and BRCAwt HGSOC patients’ tissues to identify new drug sensitivity biomarkers (Kerr et al., 2013; Lane et al., 2015; Buttarelli et al., 2022). Moreover, Cook DP et al. used syngeneic models and tumors derived from mice to evaluate the origin, epigenetic, and phenotypic differences in order to establish the most useful model for research and therapeutic tests (Cook et al., 2023).

In addition, the feasibility of chemoresistance tests in ascites cultures has been demonstrated (den Ouden et al., 2020). Unlike other studies, we are the first to comprehensively analyze the hub genes related to EM and drug resistance in CIS/DOX-treated and untreated AS cultures at the transcriptional level. In addition, we investigated two datasets that contain RNA-seq of drug-resistant cells (SKOV3 and IGROV-1).

Our *in vitro* data indicate that apoptosis markers are elevated following both treatments, while only CIS affects migration in the CIS-sensitive group. Importantly, we observed a distinct CDH1, GATA6, and KRT18 pattern in response to drug exposure. Specifically, we observed a significant increase in CDH1 and GATA6 after DOX treatment and a notable decrease after CIS treatment. In addition to this, a slight decline was observed after OHP treatment. Leung D et al. showed that CDH1 is downregulated in CARB-resistant cells, leading to enhanced cellular migration and reduced proliferation (Leung et al., 2022). Moreover GATA6 depletion correlates with the downregulation of epithelial markers (Capo-chichi et al., 2003; Martinelli et al., 2017). In contrast, KRT18 was upregulated after CIS treatment in AS and IGROV-1, while DOX treatment determined a lower gene expression. Similarly, it has been shown that KRT18 downregulation improves CIS sensitivity and EMT-independent collective migration in epithelial cancer cells (Fortier et al., 2013). This mechanism can explain the difference in the expression of KRT18 and CDH1 between the two types of treatments; KRT18 decreases and CDH1 increases after DOX treatment, resulting in unaffected migration.

Furthermore, we observed a significant increase in VIM and a decrease in KRT19 after both CIS and DOX treatment in AS. On the contrary, VIM, an intermediate filament protein that preserves cellular integrity, has been reported to decrease in drug-resistant ovarian cells. Its depletion leads to CIS resistance via the downregulation of cytoskeleton organization proteins, inducing the cancer stem cell phenotype and prolonged G2 arrest in drug-resistant cells (Kanakkanthara et al., 2012; Jin et al., 2014; Huo et al., 2016). Among these studies, Latif et al. found that VIM was upregulated at both transcriptional and post-translational levels after the CIS treatment of metastatic epithelial ovarian tumor cells, increasing stemness and drug resistance (Latifi et al., 2011). These discrepancies can rely on the phenotype of the cells treated. Indeed, the upregulation in VIM levels may be due to the treatment of mesenchymal cells. Therefore, designing a successful treatment strategy for advanced ovarian cancer requires investigation of the ascites microenvironment because it contains relevant molecules that lead to chemoresistance and disease progression. In addition to all these, the significant drawback of ovarian cancer cell lines is due to the accumulation of various genetic and phenotypic defects during years of culture, which no longer correctly reflect the clinical condition (Domcke et al., 2013).

Consistent with previous observations (Woopen et al., 2014; Communal et al., 2021; Sun et al., 2023), our analysis revealed that KRT19 and KRT7 negatively correlate with survival outcomes, while EPCAM has a positive correlation. KRT19 was reported as a potential immunotherapy target (Sun et al., 2023), and overexpression of this molecule promotes the proliferation and migration of cancer cells via Wnt/β-catenin signaling (Lu et al., 2020). In contrast, downregulation of KRT19 has been reported in tumor breast tissue compared to adjacent tissue, and it has been associated with an aggressive phenotype and chemoresistance of cancer cells (Ju et al., 2013; Saha et al., 2017; Saha et al., 2018).

At least, only VIM and GATA6 expressions have shown the same signature in tissue samples before/after CARB and PAX combination chemotherapy. Indeed, a lower expression has been obtained explicitly in the late-recurrence group, but a higher expression, after drug exposure.

To sum up, our results indicate that after DOX and CIS treatment, AS acquires an invasive-intermediate EMT state by the overexpression of both epithelial and mesenchymal markers. In both cases, VIM overexpression and KRT19 downregulation have been reported. Further research will investigate how these genes are involved in AS chemoresistance by expanding the sample size and conducting basic experiments. However, the ascites remains an exclusive and accessible sample from HGSOC patients to explore tumor progression and molecular pathways involved in chemoresistance. This could lead us to improve personalized treatment decisions by developing new targets to overcome drug resistance in HGSOC.

## Data Availability

The original contributions presented in the study are included in the article/Supplementary Material; further inquiries can be directed to the corresponding authors.
