# Supplementary material for: EM-transcriptomic signature predicts drug response in advanced stages of high-grade serous ovarian carcinoma based on ascites-derived primary cultures
Source: Front Pharmacol. 2024 Mar 6;15:1363142. doi: 10.3389/fphar.2024.1363142 (PMC10953505; doi:10.3389/fphar.2024.1363142)
Supplement: Supplementary file 1 [file DataSheet1.zip › Supplementary Figure and Table Legends_.docx]

Supplementary Figure and Table Legends

for

EM-transcriptomic signature predicts drug response in advanced stages of high-grade serous ovarian carcinoma based on ascites-derived primary cultures

Diana-Roxana Constantinescu^1†^, Andrei Sorop^1†^, Alina-Veronica Ghionescu^1†^, Daniela Lixandru^1, 2^, Vlad Herlea^1,2,3*^, Nicolae Bacalbasa^1,2,4*^, Simona Olimpia Dima^1,2,4^

^1^ Center of Excellence in Translational Medicine, Fundeni Clinical Institute, Bucharest, Romania

^2^ University of Medicine and Pharmacy “Carol Davila”, Bucharest, Romania

^3^ Department of Pathology-Fundeni Clinical Institute, Bucharest, Romania

^4^ Center of Digestive Diseases and Liver Transplantation, Fundeni Clinical Institute, Bucharest, Romania

*** Correspondence:**Nicolae Bacalbasa, MD, PhD, Fundeni Clinical Institute, 022328 Bucharest, Romania
[nicolaebacalbasa@gmail.com](mailto:nicolaebacalbasa@gmail.com)

Vlad Herlea, MD, PhD, Fundeni Clinical Institute, 022328 Bucharest, Romania

[herlea2002@gmail.com](mailto:herlea2002@gmail.com)

 ^†^These authors have contributed equally to this work and share first authorship

**Supplementary Figure Legends:**

**Supplementary Figure 1. Combination of TCGA-OV and GTEx samples and correction of batch** effects using sva R package. PCA plot of TCGA-OV and GTEx samples after correcting batch effects.

**Supplementary Figure 2. Volcano plot of the mRNA sequencing data representing differentially expressed genes** for TCGA-OV primary tumor samples versus GTEx ovarian normal samples. The data for all genes is plotted as log2 fold change versus the −log10 of the corrected p value. Thresholds are shown as lines on X-axis and Y-axis (fold change = 2.0, pCutoff = 10e-10, respectively)

**Supplementary Figure 3. Graphical diagnostic for multivariate Cox model using the function ggcoxzph** [function in survminer R package], which produces, for each parameter, graphs of the scaled Schoenfeld residuals against the transformed time. From the output, the test is not statistically significant for each of the variables, and the global test is also not statistically significant, thus we can assume that hazards are proportional at each point in time throughout follow-up. These results conclude that the proportional hazards assumption is satisfied for each parameter.

**Supplementary Table Legends:**

**Supplementary Table 1**– **Primer pairs used for RT-qPCR**

**Supplementary Table 2. Differential gene expression analysis using the Deseq2 pipeline** with default settings using TCGA-OV and GTEx RNA sequencing data (raw counts) that were combined with batch normalization using the R package “sva”(in the R Studio program).

**Supplementary Table 3. GO Enrichment Analysis of significant DEGs from TCGA-OV datasets** based on Molecular functions terms (clusterProfiler (version 4.8.2) R package)

**Supplementary Table 4. GO Enrichment Analysis of significant DEGs from TCGA-OV datasets** based on Biological processes terms (clusterProfiler (version 4.8.2) R package)

**Supplementary Table 5. GO Enrichment Analysis of significant DEGs from TCGA-OV datasets** based on Cellular Components terms (using clusterProfiler (version 4.8.2) R package)

**Supplementary Table 6. Exploring the GeneCards (https://www.genecards.org/) database** using the keywords pattern “(drug-resistant) AND (EMT) AND (keratin) AND (ovarian cancer)” we obtained 1265 drug resistance-EMT-keratin-related protein-coding genes

**Supplementary Table 7. The intersection of significant DEGs from the TCGA-OV dataset** and investigated genes from the GeneCards database

**Supplementary Table 8. EM-associated genes (CDH1, CDH2, VIM, GATA6, EPCAM, KRT7, KRT18 and KRT19)** correlated with chemoresistance in HGSOC and intersected with TCGA-GeneCards

**Supplementary Table 9. IHC score of ascites primary culture and primary tumor** from the same HGSOC patients
