# Supplementary material for: EM-transcriptomic signature predicts drug response in advanced stages of high-grade serous ovarian carcinoma based on ascites-derived primary cultures
Source: Front Pharmacol. 2024 Mar 6;15:1363142. doi: 10.3389/fphar.2024.1363142 (PMC10953505; doi:10.3389/fphar.2024.1363142)
Supplement: Supplementary file 1 [file DataSheet1.zip › Supplementary Table 1_Primer pairs used for RT-qPCR.docx]

**Supplementary Table 1– Primer pairs used for RT-qPCR**

| **Symbol** | **Forward sequence** | **Reverse sequence** | **Gene name** |
| --- | --- | --- | --- |
| **β-actin** | CTCTTCCAGCCTTCCTTCCT | AGCACTGTGTTGGCGTACAG | β-actin |
| **CDH1** | GAATGACAACAAGCCCGAAT | GACCTCCATCACAGGTTCC | Cadherin1 |
| **EPCAM** | GCTAAACTGCTTTTGAATAA | TCTCCCAAGTTTTGAGCCAT | Epithelial Cellular Adhesion Molecule |
| **CDH2** | GGTGGAGGAGAAGAAGACCAG | GCATCAGGCTCCACAGT | Cadherin2 |
| **VIM** | GAGAACTTTGCCGTTGAAGC | GCTTCCTGTAGGTGGCAATC | Vimentin |
| **KRT7** | GTTCCATTTGCAAAGGCTGT | CAGGTGGTTACCCGAAAGA | Keratin 7 |
| **KRT18** | CAGAGATCGAGGCTCTCAAGGA | GGCATCTACCTCCACGGTCAA | Keratin 18 |
| **KRT19** | TGCGGGACAAGATTCTTGGT | GCAGGACAATCCTGGAGTTCTC | Keratin 19 |
| **GATA6** | CCATTCCCATGACTCCAACT | TGAGGCTGTAGGTTGTGTTG | GATA Binding Protein 6 |
