# Supplementary material for: EM-transcriptomic signature predicts drug response in advanced stages of high-grade serous ovarian carcinoma based on ascites-derived primary cultures
Source: Front Pharmacol. 2024 Mar 6;15:1363142. doi: 10.3389/fphar.2024.1363142 (PMC10953505; doi:10.3389/fphar.2024.1363142)
Supplement: Supplementary file 1 [file DataSheet1.zip › Supplementary Table 8. EM-associated genes.docx]

**Supplementary Table 8. EM-associated genes (CDH1, CDH2, VIM, GATA6, EPCAM, KRT7, KRT18 and KRT19) correlated with chemoresistance in HGSOC and intersected with TCGA-GeneCards**

|  | Gene name | Expression (TCGA-OV) | References | Intersection with TCGA-GeneCards |
| --- | --- | --- | --- | --- |
| 1 | SNAI1 | - | (Kielbik et al., 2021); (Kielbik et al., 2023); (Hojo et al., 2018); (Wang et al., 2020) | - |
| 2 | SNAI2 | ↓ | (Yoshida et al., 2009); (Kielbik et al., 2021) | - |
| 3 | TWSIT | - | (Kielbik et al., 2021); (Yoshida et al., 2009); (Roberts et al., 2016) | - |
| 4 | ZEB1 | ↓ | (Rae et al., 2022); (An et al., 2017); (Inoue-Yamauchi and Oda, 2020) | - |
| 5 | VIM | ↓ | (Huo et al., 2016); (Usman et al., 2021) | Intersected |
| 6 | CDH1 | ↑ | (Rosso et al., 2017); (Klymenko et al., 2017); (Communal et al., 2021) | Intersected |
| 7 | CDH2 | ↑ | (Assidi, 2022); (Klymenko et al., 2017) | Intersected |
| 8 | EPCAM | ↑ | (Tayama et al., 2017); (Tavsan and Ayar Kayali, 2020) | Intersected |
| 9 | GATA6 | ↓ | (Shen et al., 2019); (Zhou et al., 2022); (Gao et al., 2023) | Intersected |
| 10 | KRT7 | ↑ | (Machino et al., 2023); (Wang et al., 2023); (Communal et al., 2021) | Intersected |
| 11 | KRT18 | ↑ | (Machino et al., 2023); (Fortier et al., 2013); (Gaytan et al., 2018) | Intersected |
| 12 | KRT19 | ↑ | (Machino et al., 2023); (Sun et al., 2023) | Intersected |
| Note: ↑overexpression ; - no changes | | | | |

AN, J., LV, W. & ZHANG, Y. 2017. LncRNA NEAT1 contributes to paclitaxel resistance of ovarian cancer cells by regulating ZEB1 expression via miR-194. *Onco Targets Ther,* 10**,** 5377-5390.

ASSIDI, M. 2022. High N-Cadherin Protein Expression in Ovarian Cancer Predicts Poor Survival and Triggers Cell Invasion. *Front Oncol,* 12**,** 870820.

COMMUNAL, L., ROY, N., CAHUZAC, M., RAHIMI, K., KOBEL, M., PROVENCHER, D. M. & MES-MASSON, A. M. 2021. A Keratin 7 and E-Cadherin Signature Is Highly Predictive of Tubo-Ovarian High-Grade Serous Carcinoma Prognosis. *Int J Mol Sci,* 22.

FORTIER, A. M., ASSELIN, E. & CADRIN, M. 2013. Keratin 8 and 18 loss in epithelial cancer cells increases collective cell migration and cisplatin sensitivity through claudin1 up-regulation. *J Biol Chem,* 288**,** 11555-71.

GAO, F., WU, Q. & LU, D. 2023. MicroRNA-10a-5p-mediated downregulation of GATA6 inhibits tumor progression in ovarian cancer. *Hum Cell*.

GAYTAN, F., MORALES, C., ROA, J. & TENA-SEMPERE, M. 2018. Changes in keratin 8/18 expression in human granulosa cell lineage are associated to cell death/survival events: potential implications for the maintenance of the ovarian reserve. *Hum Reprod,* 33**,** 680-689.

HOJO, N., HUISKEN, A. L., WANG, H., CHIRSHEV, E., KIM, N. S., NGUYEN, S. M., CAMPOS, H., GLACKIN, C. A., IOFFE, Y. J. & UNTERNAEHRER, J. J. 2018. Snail knockdown reverses stemness and inhibits tumour growth in ovarian cancer. *Sci Rep,* 8**,** 8704.

HUO, Y., ZHENG, Z., CHEN, Y., WANG, Q., ZHANG, Z. & DENG, H. 2016. Downregulation of vimentin expression increased drug resistance in ovarian cancer cells. *Oncotarget,* 7**,** 45876-45888.

INOUE-YAMAUCHI, A. & ODA, H. 2020. EMT-inducing transcription factor ZEB1-associated resistance to the BCL-2/BCL-X(L) inhibitor is overcome by BIM upregulation in ovarian clear cell carcinoma cells. *Biochem Biophys Res Commun,* 526**,** 612-617.

KIELBIK, M., PRZYGODZKA, P., SZULC-KIELBIK, I. & KLINK, M. 2023. Snail transcription factors as key regulators of chemoresistance, stemness and metastasis of ovarian cancer cells. *Biochim Biophys Acta Rev Cancer,* 1878**,** 189003.

KIELBIK, M., SZULC-KIELBIK, I. & KLINK, M. 2021. Impact of Selected Signaling Proteins on SNAIL 1 and SNAIL 2 Expression in Ovarian Cancer Cell Lines in Relation to Cells' Cisplatin Resistance and EMT Markers Level. *Int J Mol Sci,* 22.

KLYMENKO, Y., KIM, O., LOUGHRAN, E., YANG, J., LOMBARD, R., ALBER, M. & STACK, M. S. 2017. Cadherin composition and multicellular aggregate invasion in organotypic models of epithelial ovarian cancer intraperitoneal metastasis. *Oncogene,* 36**,** 5840-5851.

MACHINO, H., DOZEN, A., KONAKA, M., KOMATSU, M., NAKAMURA, K., IKAWA, N., SHOZU, K., ASADA, K., KANEKO, S., YOSHIDA, H., KATO, T., NAKAYAMA, K., SALOURA, V., KYO, S. & HAMAMOTO, R. 2023. Integrative analysis reveals early epigenetic alterations in high-grade serous ovarian carcinomas. *Exp Mol Med,* 55**,** 2205-2219.

RAE, S., SPILLANE, C., BLACKSHIELDS, G., MADDEN, S. F., KEENAN, J. & STORDAL, B. 2022. The EMT-activator ZEB1 is unrelated to platinum drug resistance in ovarian cancer but is predictive of survival. *Hum Cell,* 35**,** 1547-1559.

ROBERTS, C. M., TRAN, M. A., PITRUZZELLO, M. C., WEN, W., LOEZA, J., DELLINGER, T. H., MOR, G. & GLACKIN, C. A. 2016. TWIST1 drives cisplatin resistance and cell survival in an ovarian cancer model, via upregulation of GAS6, L1CAM, and Akt signalling. *Sci Rep,* 6**,** 37652.

ROSSO, M., MAJEM, B., DEVIS, L., LAPYCKYJ, L., BESSO, M. J., LLAURADO, M., ABASCAL, M. F., MATOS, M. L., LANAU, L., CASTELLVI, J., SANCHEZ, J. L., PEREZ BENAVENTE, A., GIL-MORENO, A., REVENTOS, J., SANTAMARIA MARGALEF, A., RIGAU, M. & VAZQUEZ-LEVIN, M. H. 2017. E-cadherin: A determinant molecule associated with ovarian cancer progression, dissemination and aggressiveness. *PLoS One,* 12**,** e0184439.

SHEN, W., NIU, N., LAWSON, B., QI, L., ZHANG, J., LI, T., ZHANG, H. & LIU, J. 2019. GATA6: a new predictor for prognosis in ovarian cancer. *Hum Pathol,* 86**,** 163-169.

SUN, Z., ZHOU, R., DAI, J., CHEN, J., LIU, Y., WANG, M., ZHOU, R., LIU, F., ZHANG, Q., XU, Y. & ZHANG, T. 2023. KRT19 is a Promising Prognostic Biomarker and Associates with Immune Infiltrates in Serous Ovarian Cystadenocarcinoma. *Int J Gen Med,* 16**,** 4849-4862.

TAVSAN, Z. & AYAR KAYALI, H. 2020. EpCAM-claudin-tetraspanin-modulated ovarian cancer progression and drug resistance. *Cell Adh Migr,* 14**,** 57-68.

TAYAMA, S., MOTOHARA, T., NARANTUYA, D., LI, C., FUJIMOTO, K., SAKAGUCHI, I., TASHIRO, H., SAYA, H., NAGANO, O. & KATABUCHI, H. 2017. The impact of EpCAM expression on response to chemotherapy and clinical outcomes in patients with epithelial ovarian cancer. *Oncotarget,* 8**,** 44312-44325.

USMAN, S., WASEEM, N. H., NGUYEN, T. K. N., MOHSIN, S., JAMAL, A., TEH, M. T. & WASEEM, A. 2021. Vimentin Is at the Heart of Epithelial Mesenchymal Transition (EMT) Mediated Metastasis. *Cancers (Basel),* 13.

WANG, Q., LOPEZ-OZUNA, V. M., BALOCH, T., BITHRAS, J., AMIN, O., KESSOUS, R., KOGAN, L., LASKOV, I. & YASMEEN, A. 2020. Biguanides in combination with olaparib limits tumorigenesis of drug-resistant ovarian cancer cells through inhibition of Snail. *Cancer Med,* 9**,** 1307-1320.

WANG, S., LI, H., LI, M., LIU, X., YU, S., HUANG, H. & WANG, X. 2023. Role of the KRT7 Biomarker in Immune Infiltration and Paclitaxel Resistance in Ovarian. *Altern Ther Health Med,* 29**,** 132-140.

YOSHIDA, J., HORIUCHI, A., KIKUCHI, N., HAYASHI, A., OSADA, R., OHIRA, S., SHIOZAWA, T. & KONISHI, I. 2009. Changes in the expression of E-cadherin repressors, Snail, Slug, SIP1, and Twist, in the development and progression of ovarian carcinoma: the important role of Snail in ovarian tumorigenesis and progression. *Med Mol Morphol,* 42**,** 82-91.

ZHOU, Q., YANG, H. J., ZUO, M. Z. & TAO, Y. L. 2022. Distinct expression and prognostic values of GATA transcription factor family in human ovarian cancer. *J Ovarian Res,* 15**,** 49.
