# Supplementary material for: EM-transcriptomic signature predicts drug response in advanced stages of high-grade serous ovarian carcinoma based on ascites-derived primary cultures
Source: Front Pharmacol. 2024 Mar 6;15:1363142. doi: 10.3389/fphar.2024.1363142 (PMC10953505; doi:10.3389/fphar.2024.1363142)
Supplement: Supplementary file 1 [file DataSheet1.zip › Supplementary Table 9_IHC score of ascites primary culture and primary tumor from the same HGSOC patients.docx]

| **Supplementary Table 9. IHC score of ascites primary culture and primary tumor from the same HGSOC patients** | | | | | | |
| --- | --- | --- | --- | --- | --- | --- |
| Sample ID | Sample type | E Cadherin/CDH1 IHC score: 0=absent; 1=1-10% positive cells; 2=10-25% positive cells; 3= >25% positive cells | N Cadherin/CDH2 IHC score: 0=absent; 1=1-10% positive cells; 2=10-25% positive cells; 3= >25% positive cells | KRT18 IHC score: 0=absent; 1=1-10% positive cells; 2=10-25% positive cells; 3= >25% positive cells | KRT19 IHC score: 0=absent; 1=1-10% positive cells; 2=10-25% positive cells; 3= >25% positive cells | KRT7 IHC score: 0=absent; 1=1-10% positive cells; 2=10-25% positive cells; 3= >25% positive cells |
| AS23 | primary tumor cell culture derived from ascites | 3 | 1 | 3 | 2 | 3 |
| T23 | primary ovary tumor tissue | 3 | 3 | 3 | 2 | 1 |
| AS53 | primary tumor cell culture derived from ascites | 3 | 2 | 3 | 3 | 3 |
| T53 | primary ovary tumor tissue | 2 | 3 | 3 | 3 | 3 |
| AS77 | primary tumor cell culture derived from ascites | 3 | 1 | 3 | 3 | 3 |
| T77 | primary ovary tumor tissue | 3 | 2 | 3 | 3 | 3 |
| A83 | primary tumor cell culture derived from ascites | 3 | 2 | 3 | 3 | 3 |
| T83 | primary ovary tumor tissue | 3 | 3 | 3 | 3 | 3 |
