# Supplementary figures and images for: EM-transcriptomic signature predicts drug response in advanced stages of high-grade serous ovarian carcinoma based on ascites-derived primary cultures
Source: Front Pharmacol. 2024 Mar 6;15:1363142. doi: 10.3389/fphar.2024.1363142 (PMC10953505; doi:10.3389/fphar.2024.1363142)

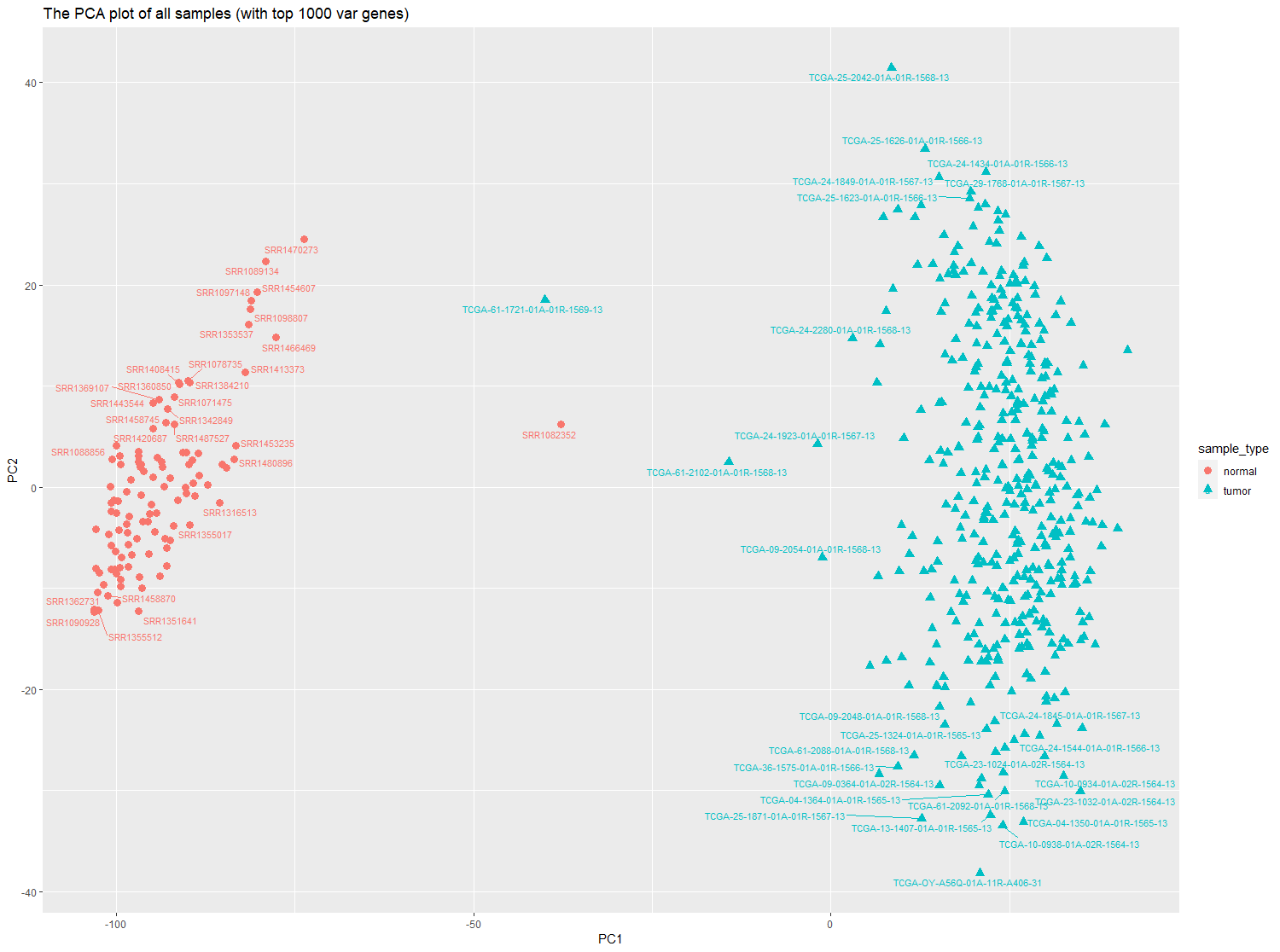

Supplement: Supplementary file 1 [file DataSheet1.zip › Supplementary Figure 1.tiff]

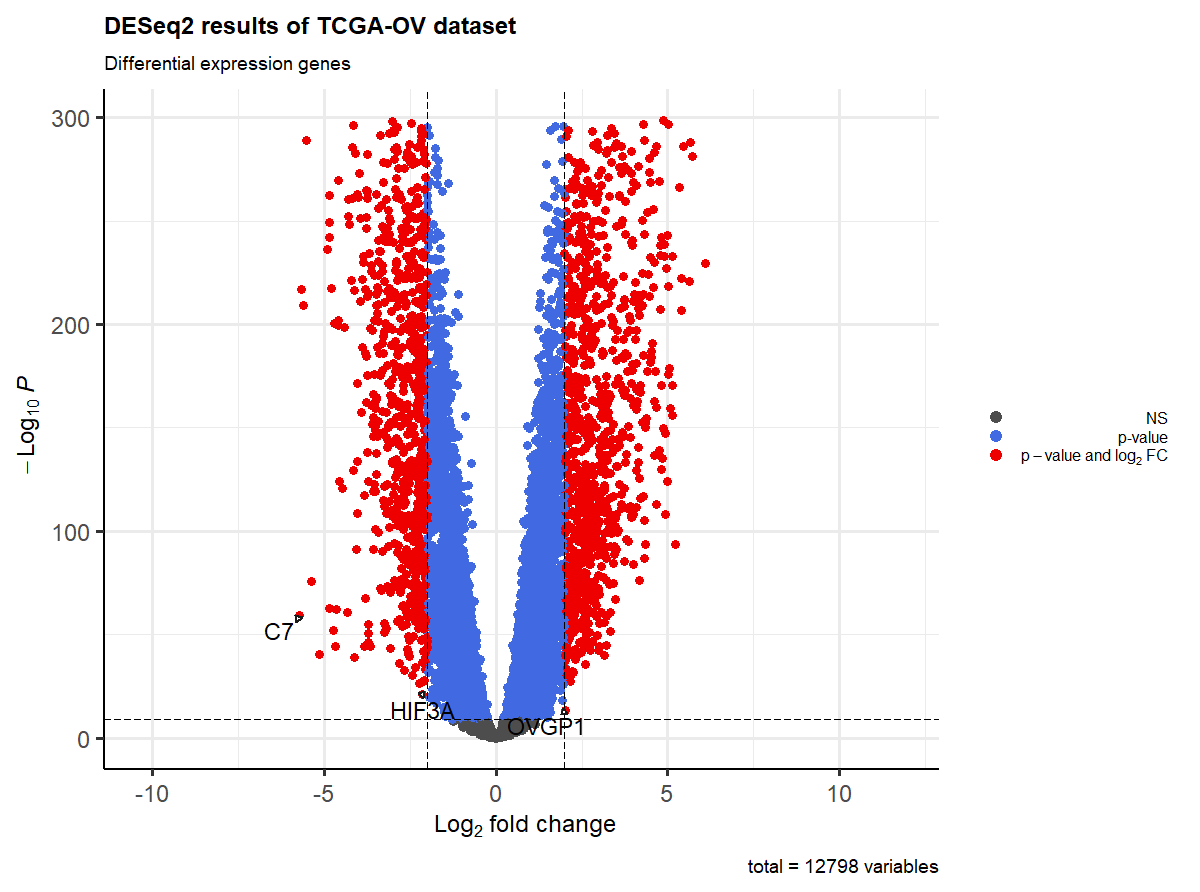

Supplement: Supplementary file 1 [file DataSheet1.zip › Supplementary Figure 2.tiff]

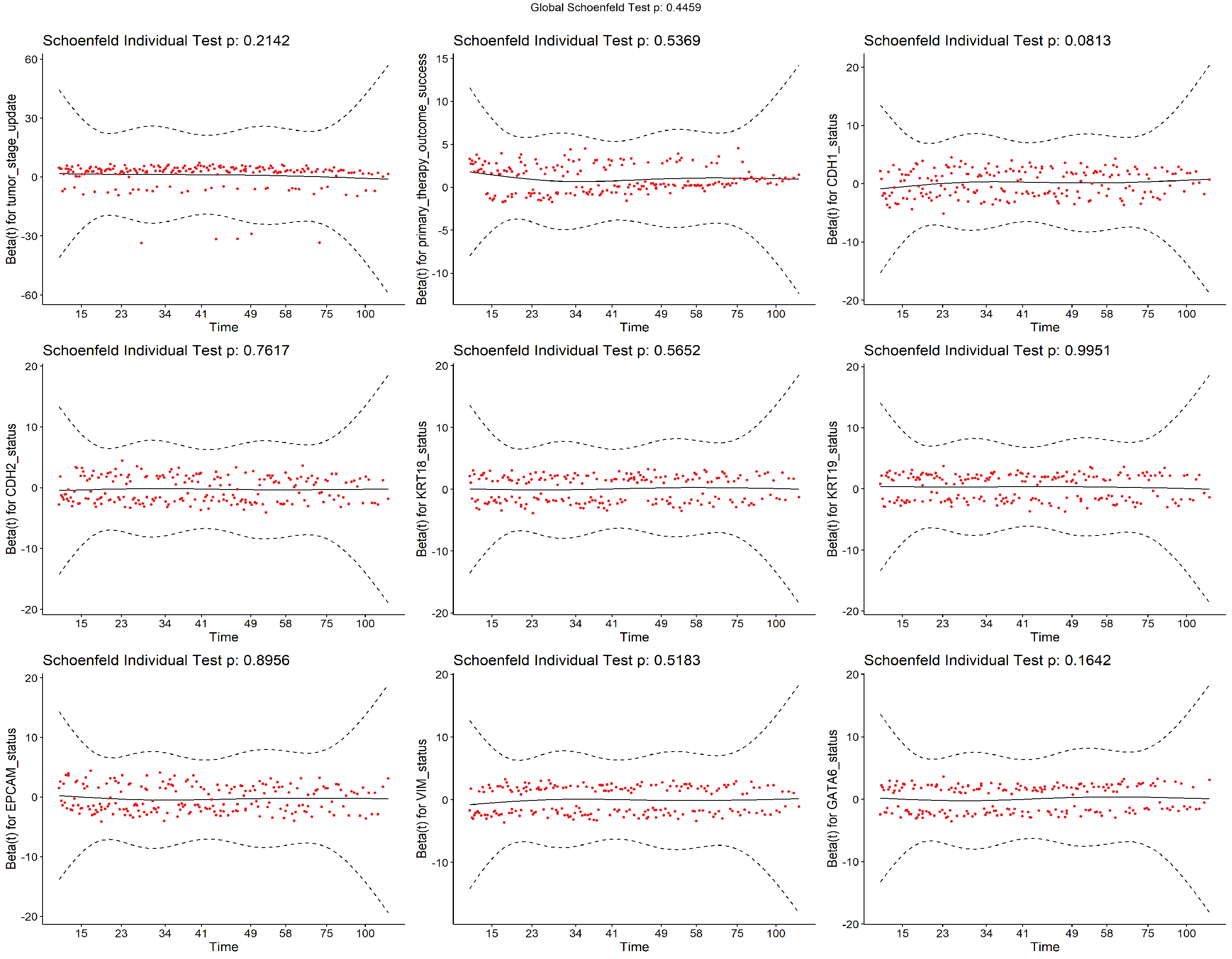

Supplement: Supplementary file 1 [file DataSheet1.zip › Supplementary Figure 3.tiff]
